# Vitamin D as a cellular endocrine system: Tissue-specific microcircuits, immune reprogramming, and metabolic resistance

**DOI:** 10.1016/j.isci.2026.116160

**Published:** 2026-06-01

**Authors:** Jialiang Feng, Jun Xiao, Zhiyuan Gao, Hui Zhou, Xilian Li, Yini Tian, Biao Gao

**Affiliations:** 1 Neurology Department, Shidong Hospital, Yangpu District, Shanghai, China; 2 Clinical Laboratory Medicine Center, Yueyang Hospital of Integrated Traditional Chinese and Western Medicine, Shanghai University of Traditional Chinese Medicine, Shanghai, China; 3 Obstetrics & Gynecology Hospital of Fudan University, Shanghai Key Lab of Reproduction and Development, Shanghai Key Lab of Female Reproductive Endocrine Related Diseases, Shanghai, China; 4 Faculty of Naval Medicine, Naval Medical University, Shanghai, China; 5 Teaching and Research Support Center, Naval Medical University, Shanghai, China

**Keywords:** biological sciences

## Abstract

Circulating 25-hydroxyvitamin D is the standard marker of vitamin D status, but tissue responses to vitamin D vary across organs, inflammatory states, and clinical settings. This review frames vitamin D biology as a cellular endocrine system in which substrate transport, local CYP27B1-mediated activation, CYP24A1-mediated inactivation, and VDR responsiveness form tissue-specific microcircuits. Evidence from barrier epithelia, immune cells, and brain-resident cells indicates that megalin/cubilin-dependent uptake, local enzyme balance, redox state, and downstream transcriptional competence can decouple serum 25-hydroxyvitamin D from intracellular vitamin D activity. We use metabolic vitamin D resistance to describe acquired low-responsiveness states driven by inflammation, aging, redox stress, or chronic disease. This framework clarifies biomarker interpretation and supports more context-aware approaches to supplementation, trial design, and tissue-targeted intervention.

## Graphical abstract

**Figure:**
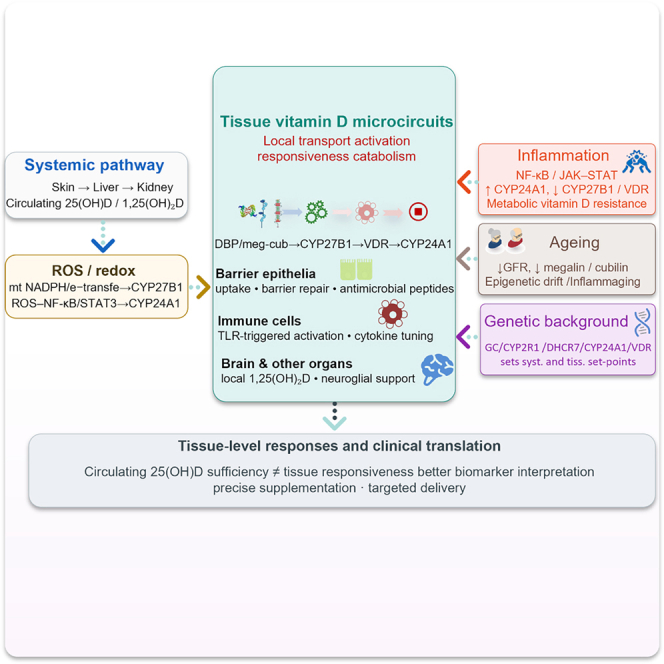

## Introduction

Traditional textbook models depict vitamin D metabolism as a linear endocrine cascade: vitamin D_3_ synthesized in the skin or obtained from the diet is 25-hydroxylated in the liver to 25-hydroxyvitamin D [25(OH)D], which is subsequently converted in the renal proximal tubule by 1α-hydroxylase (CYP27B1) into the active hormone 1,25-dihydroxyvitamin D [1,25(OH)_2_D].^1^^,^^2^^,^^3^^,^^4^ Within this framework, serum 25(OH)D has traditionally been used as the principal circulating indicator of systemic vitamin D supply, and clinical practice has been organized around universal concentration thresholds and largely fixed-dose supplementation strategies.

Yet, a persistent paradox remains. Broad overviews have repeatedly highlighted the limits of treating vitamin D as a single circulating hormone, particularly when interpreting pleiotropic, non-skeletal outcomes.^5^^,^^6^^,^^7^ Across large observational cohorts, lower 25(OH)D levels are associated with higher risk of cardiovascular disease, immune dysregulation, and all-cause mortality, whereas randomized supplementation trials have generally yielded modest or null benefits, especially for non-skeletal endpoints.^8^^,^^9^^,^^10^^,^^11^ These trials are also structurally prone to null findings because vitamin D is a nutrient rather than a conventional drug: many enroll participants who are already vitamin D sufficient, permit background intake from diet or supplements, and, for ethical reasons, may not maintain a truly vitamin D-unexposed control condition. Even within trials, responders and non-responders coexist across seemingly similar baseline strata, a pattern often interpreted as metabolic vitamin D resistance, differential tissue uptake, or context-dependent biology.^12^^,^^13^ These discordances may be better framed as a redox-sensitive microcircuit problem: inflammatory signaling and oxidative stress can re-tune local activation, catabolism, and downstream receptor-dependent responsiveness, thereby uncoupling systemic 25(OH)D supply from intracellular exposure and transcriptional output.^14^^,^^15^

These inconsistencies raise a fundamental question: is circulating 25(OH)D sufficient to capture the true “exposure” and functional state of vitamin D in each target tissue? Experimental work has shown that multiple extra-renal cell types—including keratinocytes, monocytes/macrophages, epithelial barrier cells, glial cells, and endothelial cells—express CYP27B1, CYP24A1, and the vitamin D receptor (VDR), and thus possess the capacity to locally convert 25(OH)D into active and inactive metabolites.^16^^,^^17^ In addition, factors such as uptake of vitamin D-binding protein (DBP)-25(OH)D complexes via megalin/cubilin-mediated endocytosis,^18^^,^^19^^,^^20^ tissue-specific enzyme expression profiles, and intracellular substrate partitioning can decouple the pool of “locally available substrate” from the total circulating concentration,^21^ making serum 25(OH)D resemble more an upstream supply signal than a direct surrogate for tissue exposure.

Against this backdrop, the concepts of “intracrine” signaling and “local microcircuits” have emerged. From this perspective, target cells are not passive recipients of a uniform hormonal input from the circulation. Rather, by combining receptor-mediated transport of DBP-25(OH)D, the local balance between CYP27B1 and CYP24A1, and VDR-dependent transcriptional programs, cells assemble spatiotemporally specialized vitamin D microcircuits that are tuned to their functional context. Distinct combinations of these microcircuits can coexist within the same organ, creating heterogeneous “exposure-response units” in which vitamin D signaling differs across microenvironments such as inflammation, ischemia, regeneration, or tumor niches. Experimental and clinical observations further suggest that, under sustained inflammatory or chronic disease conditions, some tissues develop an acquired low-responsiveness state characterized by persistent CYP24A1 upregulation, reduced effective VDR signaling, altered CYP27B1 activity, or combinations thereof. In this review, we use the term metabolic vitamin D resistance as an operational label for this tissue state, in which adequate or near-adequate circulating 25(OH)D fails to translate into proportionate intracellular vitamin D activity because local catabolism, impaired receptor responsiveness, or maladaptive microcircuit remodeling outweigh substrate supply. Such phenotypes have been reported in intestinal mucosa, alveolar epithelium, placenta, and selected central nervous system models, although their magnitude, mechanisms, and reversibility vary by tissue and disease context.^7^^,^^22^^,^^23^^,^^24^^,^^25^

Despite the rapid growth of this literature, an integrated framework that links vitamin D biology across the system-organ-cell-subcellular hierarchy remains underdeveloped. In this review, we advance three related propositions. First, vitamin D biology is better understood as a tissue-specific cellular endocrine system composed of local transport-metabolism-receptor microcircuits rather than as a uniform endocrine axis. Second, immune and inflammatory signaling do not merely intersect with these microcircuits but can progressively reprogram them toward a catabolism-dominant, low-responsiveness state that we term metabolic vitamin D resistance. Third, this framework helps explain why circulating 25(OH)D alone often incompletely predicts tissue activity, clinical phenotype, or supplementation response. On this basis, we integrate transport mechanisms, metabolic enzyme networks, tissue-specific microcircuits, and their remodeling by inflammation, aging, and genetic background into a unified model intended to bridge basic biology with clinical and public health interpretation. The operational definitions of the core concepts used throughout this review are summarized in Box 1.

Box 1 **Core operational concepts used in this review**

Cellular endocrine system: a distributed model of vitamin D biology in which multiple non-renal tissues possess the machinery required to regulate local vitamin D activation, inactivation, and response according to tissue context.

Vitamin D microcircuits (intracrine microcircuits): spatially confined regulatory units comprising substrate delivery, local activation by CYP27B1, signal transduction through VDR, and catabolism by CYP24A1.

Metabolic vitamin D resistance: an acquired tissue state in which adequate or near-adequate circulating 25(OH)D fails to produce proportionate intracellular vitamin D activity because local catabolism, impaired receptor responsiveness, or maladaptive microcircuit remodeling predominates.

### Molecular architecture: Precise assembly of microcircuits

#### Conceptual framework and core components

The cellular intracrine microcircuits of vitamin D are highly hierarchical. They integrate local metabolic and signaling networks within the cell and are subject to tight tissue-specific regulation. At the molecular level, this self-regulating, multi-component network can be decomposed into three core modules:

Transport module: In circulation, vitamin D and its metabolites are predominantly bound to DBP (encoded by the group-specific component gene, GC), with a smaller fraction bound to albumin and only a minute proportion in a free form. Entry into cells occurs via several routes: passive diffusion of free or loosely bound species; receptor-mediated endocytosis of DBP–vitamin D complexes through the megalin/cubilin system; and other putative transporters that have been proposed but remain poorly defined. The functional evidence and quantitative contribution of each pathway still need to be clarified by prospective, tissue-specific studies to define their relative weights and regulatory determinants.^1^^,^^20^^,^^26^^,^^27^^,^^28^^,^^29^

Metabolic module: local metabolism is driven by two cytochrome P450 enzymes. CYP27B1 (25-hydroxyvitamin D-1α-hydroxylase) catalyzes the conversion of 25(OH)D to 1,25(OH)_2_D and depends on the mitochondrial electron transport chain for its activity, whereas CYP24A1 (25-hydroxyvitamin D-24-hydroxylase) mediates sequential hydroxylation at the C-24 and C-23 positions, degrading active metabolites to water-soluble end-products (such as calcitroic acid) to terminate signaling and maintain metabolic homeostasis.^1^^,^^30^^,^^31^ Recent studies have suggested that mitochondrial and non-mitochondrial targeting variants of CYP24A1, generated by alternative splicing, may extend the metabolic network beyond the classical C-24/23 pathways and create a series of intermediate metabolites that are not yet fully characterized. Experimental data across multiple tissues support local CYP27B1 activity and CYP24A1-mediated degradation routes, and the formation of mitochondrial-associated metabolic microdomains provides a spatial dimension tonbsp;vitamin D signaling.^32^^,^^33^^,^^34^^,^^35^ Systematic summaries of the CYP24A1 substrate spectrum and the physiological roles of its products have also been published.^35^^,^^36^^,^^37^

Signal-transduction module: The VDR a ligand-activated nuclear receptor that typically heterodimerizes with retinoid X receptor (RXR), binds vitamin D response elements (VDREs), and recruits co-regulatory complexes that support target-gene transactivation.^38^^,^^39^ However, vitamin D signaling through VDR is not limited to transcriptional activation. Genomic and gene-expression studies indicate that a substantial proportion of vitamin D-responsive genes are downregulated rather than induced.^39^^,^^40^ These repressive effects appear to arise largely through indirect and context-dependent mechanisms—including altered availability of co-regulators, interference with other transcription-factor networks, and cell-specific chromatin accessibility—rather than through a single canonical VDRE-driven pathway.^39^^,^^40^^,^^41^ Functionally, this tissue specificity is illustrated by the fact that in monocytes/macrophages, vitamin D signaling is closely linked to antimicrobial peptide induction after TLR activation, whereas in intestinal epithelium it is more closely tied to tight-junction integrity and mucosal repair; in chronically inflamed or neoplastic tissues, increased CYP24A1 expression may truncate local signaling despite apparently adequate substrate availability. Accordingly, the downstream transcriptional output of vitamin D signaling through VDR differs across immune, epithelial, renal, and neural cells depending on lineage, inflammatory state, and epigenetic context.

#### Spatiotemporal dynamics and subcellular localization

Endocytosis mediated by the megalin/cubilin complex is an established route for uptake of DBP-bound vitamin D metabolites in selected epithelial tissues.^20^^,^^28^^,^^42^ After internalization, endosomal acidification is thought to facilitate release of vitamin D metabolites from DBP-containing carrier complexes. Although this dissociation process is well established in megalin/cubilin-expressing epithelia, the downstream intracellular routing of released metabolites likely differs between proximal tubular cells, placental trophoblasts, and other barrier epithelia, and remains incompletely resolved outside the kidney.^20^^,^^28^^,^^42^ Released metabolites may subsequently undergo recycling, transcellular movement, or local metabolic processing, but the relative contribution of these pathways appears to be tissue-dependent. Whether Rab11-dependent recycling is functionally important for repeated apical availability of megalin/cubilin in vitamin D metabolite uptake remains to be demonstrated directly in tissue-specific systems.^28^

CYP27B1 is predominantly localized to the matrix side of the inner mitochondrial membrane and is positioned to couple localnbsp;vitamin D activation to mitochondrial redox and electron-transfer systems.^1^^,^^32^ By contrast, CYP24A1 appears to show more context-dependent subcellular distribution, potentially influenced by splice variants, cell lineage, and metabolic state.^32^^,^^33^^,^^38^^,^^43^ Current evidence suggests that such localization may help shape local catabolic flux, but direct proof that it creates functionally discrete vitamin D microdomains remains limited. Accordingly, although spatial coupling between vitamin D metabolism and mitochondrial organization is increasingly recognized, stronger functional studies are still needed before these putative microdomains can be assigned a uniform physiological role across tissues.^32^^,^^33^^,^^43^

### Transport systems: Key gatekeepers from the circulation to the cell

#### Molecular diversity of the DBP/GC system

Vitamin DBP, encoded by the GC gene on chromosome 4q13.3, is not merely a passive carrier but a key determinant of vitamin D bioavailability. Common single-nucleotide polymorphisms (SNPs) give rise to three major electrophoretic variants—GC1F, GC1S, and GC2—which differ in glycosylation pattern, plasma concentration, and binding kinetics for 25(OH)D.^26^^,^^44^^,^^45^^,^^46^

Among these polymorphisms, the missense variants rs7041 and rs4588 are particularly functionally important, as they alter DBP conformational stability and ligand affinity.^47^^,^^48^ Cohort and genetic epidemiology studies consistently show that carriers of these alleles have lower circulating DBP concentrations and possibly altered 25(OH)D binding, thereby indirectly modifying the proportion of free and bioavailable 25(OH)D.^26^^,^^46^^,^^47^ The frequencies of these GC variants differ substantially across ancestral groups and are thought to underlie, at least in part, systematic population-level differences in vitamin D status.^49^^,^^50^^,^^51^

#### Expanded roles of the megalin-cubilin system beyond the kidney

Megalin (LRP2, ∼600 kDa) and cubilin (∼460 kDa) have long been regarded as an endocytic receptor complex specifically expressed in the brush border of renal proximal tubular cells, where they mediate the retrieval and degradation of filtered proteins. Megalin possesses a large extracellular domain composed of multiple ligand-binding clusters that recognize a broad spectrum of ligands, and a cytoplasmic tail containing several NPXY-like motifs that recruit clathrin and adaptor proteins to couple endocytosis with downstream signaling.^52^^,^^53^^,^^54^ Cubilin lacks a transmembrane domain and is anchored to the apical membrane via amnionless (AMN) to form the CUBAM complex. Its extracellular region consists of EGF-like repeats and approximately 27 CUB domains, providing a modular platform for high-affinity ligand recognition.^53^^,^^55^^,^^56^ DBP-25(OH)D complexes bind with high affinity to specific CUB domains on cubilin and are internalized through megalin-cubilin-dependent endocytosis, enabling efficient reclamation and recycling of vitamin D carrier-ligand complexes from the filtrate.^18^

Although expression of the megalin/cubilin system is most abundant in renal proximal tubular epithelial cells, later studies have demonstrated key roles in a range of extra-renal tissues.^28^ (1) In syncytiotrophoblasts of the placenta, megalin and cubilin are implicated in the transfer of maternal DBP-bound vitamin D metabolites to the fetus and may influence fetal skeletal and immune development.^57^^,^^58^^,^^59^ (2) In parathyroid chief cells, megalin expression has been reported and proposed to participate in fine-tuning local sensing of calcium-phosphate signals and the regulation of PTH secretion,^28^^,^^60^ although the functional significance of this pathway remains to be fully established. In renal proximal tubules, megalin also mediates receptor-dependent endocytosis and lysosomal degradation of parathyroid hormone (PTH), thereby contributing to the fine regulation of phosphate reabsorption and mineral homeostasis in the kidney.^61^^,^^62^ (3) In mammary epithelial cells during pregnancy and lactation, megalin/cubilin expression supports local conversion of precursors to 1,25(OH)_2_D and has been hypothesized to contribute to mammary differentiation and the precise control of vitamin D content in breast milk.^20^^,^^63^ (4) In selected neuronal subpopulations and choroid plexus epithelium, experimental data suggest that microglia-derived DBP can interact with neuronal megalin, triggering downstream signaling events that lead to synaptic injury and depression-like behaviors, thereby linking brain vitamin D carrier proteins to neural circuit homeostasis.^64^^,^^65^^,^^66^

**Figure 1:**
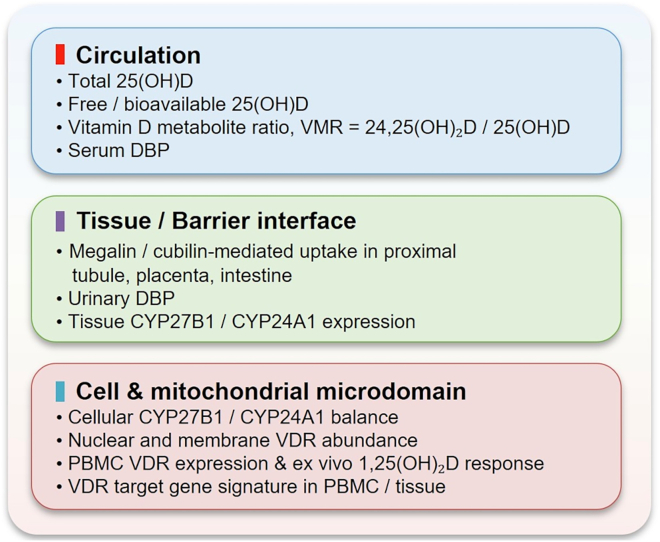
Multi-scale overview of the vitamin D cellular endocrine system The schematic depicts the vitamin D cellular endocrine system from circulation to tissue, cell, and mitochondrial microdomains. In the circulation, total 25(OH)D, free/bioavailable 25(OH)D, vitamin D metabolite ratio (VMR = 24,25(OH)_2_D/25(OH)D), and serum DBP provide complementary information on systemic substrate supply. At the blood-tissue interface, megalin/cubilin-mediated uptake in barrier epithelia (proximal tubule, placenta, intestinal epithelium) and urinary DBP reflect tissue-specific transport and proximal tubular integrity. Within target cells, CYP27B1/CYP24A1 expression, VDR abundance, and canonical VDR target gene signatures in PBMCs and tissue biopsies represent candidate readouts of local activation/inactivation balance and downstream responsiveness. Solid labels indicate clinically available assays; dashed labels denote exploratory microcircuit-level markers currently restricted to research settings. Created by the authors; not adapted from previously published material.

Taken together, these findings indicate that the vitamin D cellular endocrine system can be conceptualized along a longitudinal axis from the circulation, through barrier interfaces and tissue compartments, down to cellular and mitochondrial metabolic microdomains. Each level corresponds to distinct, and in principle measurable, biomarkers (Figure 1).

#### Revisiting the free hormone hypothesis

The “free hormone hypothesis” posits that only unbound hormone molecules are biologically active.^44^^,^^67^ Several lines of evidence support the applicability of this concept to vitamin D. (1) Free vitamin D can enter most cell types by passive diffusion.^68^ (2) In certain clinical settings, such as cirrhosis and pregnancy, free 25(OH)D concentrations appear to better reflect vitamin D status than total 25(OH)D.^69^^,^^70^ (3) Mice lacking DBP maintain normal calcium homeostasis when provided with adequate vitamin D supplementation, despite extremely low total serum concentrations of vitamin D metabolites.^71^ (4) In observational studies, free and bioavailable vitamin D often show stronger correlations with bone mineral density than total vitamin D or DBP-bound fractions.^72^

However, the megalin-cubilin system adds an essential layer of complexity to this framework. In tissues expressing this receptor complex, DBP-bound vitamin D metabolites can enter cells via receptor-mediated endocytosis with much greater efficiency than the free form can via passive diffusion.^28^ Quantitative studies in proximal tubules indicate that uptake of DBP-25(OH)D complexes is markedly more efficient than that of free 25(OH)D.^73^^,^^74^ Disruption or inhibition of megalin/cubilin leads to urinary loss of DBP-25(OH)D, limits substrate supply for local 1α-hydroxylase, and is associated with skeletal abnormalities and disordered mineral metabolism.^18^^,^^61^

Overall, the free hormone hypothesis remains a valid approximation for explaining vitamin D entry into most cell types that do not highly express megalin/cubilin. In tissues with abundant expression of this receptor system, receptor-mediated endocytosis of DBP-25(OH)D provides a parallel substrate-delivery route that can significantly shape local exposure and signaling. These two mechanisms are not mutually exclusive; instead, they form a continuum whose relative contributions are determined by tissue type, inflammatory milieu, and systemic hormone levels. On the basis of current evidence, the quantitative balance between these pathways has yet to be rigorously defined.

### Metabolic enzyme system: finely tuned balance between activation and inactivation

#### CYP27B1: From a “kidney-specific” enzyme to a widely distributed catalyst

CYP27B1 was once regarded as a “kidney-specific” enzyme confined to proximal tubular cells. Subsequent histological, transcriptomic, and functional studies have, however, demonstrated that CYP27B1 is widely expressed across multiple organs and contributes to local autocrine and paracrine regulation of vitamin D signaling.^17^^,^^21^^,^^75^

Immune system: monocytes and macrophages upregulate CYP27B1 in response to pathogen-associated molecular patterns (PAMPs) that activate Toll-like receptors (TLRs, such as TLR2/1 and TLR4). This induction is mediated via NF-κB and related inflammatory pathways, enabling these cells to convert circulating 25(OH)D into 1,25(OH)_2_D locally and to couple antimicrobial responses with vitamin D-dependent transcriptional programs.^22^^,^^25^^,^^76^ Beyond direct TLR triggering, however, immune CYP27B1 expression is also shaped by the surrounding cytokine milieu: inflammatory signals help determine whether local vitamin D activation is amplified, sustained, or counterbalanced by catabolic and receptor-level feedback, indicating that immune vitamin D metabolism is embedded in broader cytokine networks rather than governed by a single receptor pathway alone.^22^^,^^25^^,^^76^ Dendritic cells (DCs), activated T lymphocytes, and B cells can also express CYP27B1, suggesting that adaptive immune cells are capable of context-dependent local vitamin D metabolism once appropriately activated.^25^ Notably, in human CD4^+^ T cells, complement-dependent signaling has been shown to induce both CYP27B1 and VDR, enabling local vitamin D activation and promoting a transition toward self-limiting IL-10-associated responses, thereby directly linking immune activation to anti-inflammatory feedback within the vitamin D microcircuit.^77^

Epithelial barrier sites, including the intestinal and respiratory mucosa and the epidermis, CYP27B1 expression has been documented in epithelial cells and keratinocytes, where it contributes to barrier integrity and modulates local inflammation. In inflammatory bowel disease (IBD), altered CYP27B1 expression in colonic epithelial cells has been associated with impaired barrier function and heightened mucosal inflammation, supporting the concept of a “vitamin D-sensitive” epithelial microcircuit in the gut.^78^^,^^79^^,^^80^^,^^81^ In colorectal and breast tumor models, reduced CYP27B1 expression together with increased CYP24A1 expression has been associated with lower local availability of active vitamin D metabolites, weaker antiproliferative signaling, and reduced responsiveness to vitamin D-based interventions.^34^^,^^43^^,^^82^

Endocrine tissues: CYP27B1, VDR, and CYP24A1 are expressed in the parathyroid gland, and their expression patterns vary with age and calcium-phosphate status. These findings support a role for local vitamin D metabolism in fine-tuning PTH secretion via a feedback loop within the gland.^83^ In pancreatic β cells, the CYP27B1-VDR axis has been linked to glucose sensing, insulin secretion and β-cell survival, and is often downregulated or functionally impaired in the setting of type 2 diabetes and obesity.^84^ At the maternal-fetal interface, trophoblast and decidual cells show marked CYP27B1 expression that increases during early pregnancy and is considered a key source of local 1,25(OH)_2_D generation and immune tolerance. This expression is finely regulated by inflammatory mediators and endocrine signals, linking vitamin D microcircuits to placental function and pregnancy outcomes.^85^^,^^86^

Central nervous system: neurons and glial cells express CYP27B1 and CYP24A1, and their expression is dynamically altered in the context of neuroinflammation and demyelinating lesions.^87^^,^^88^ In multiple sclerosis lesions, activated microglia and astrocytes show increased CYP27B1 expression, whereas changes in VDR and CYP24A1 expression correlate with the degree of local inflammation.^88^ Pathological and confocal immunofluorescence studies in Parkinson’s disease and other neurodegenerative conditions have identified a subset of CYP27B1-positive reactive astrocytes in affected regions; these cells appear to engulf and degrade α-synuclein aggregates and to localize near relatively preserved dopaminergic neurons, and have therefore been interpreted as a putative neuroprotective or compensatory response to protein aggregation and oxidative stress.^89^^,^^90^ Taken together, these observations support the view that “brain-local vitamin D metabolism” is an integral component of neurodevelopment, synaptic homeostasis, and the pathobiology of neurodegenerative disease.^91^

#### CYP24A1 as a determinant of signal duration and local catabolic flux

CYP24A1 has traditionally been viewed as the key enzyme in the inactivation pathway of vitamin D, catalyzing the conversion of 25(OH)D and 1,25(OH)_2_D into a series of hydroxylated metabolites and thereby preventing vitamin D intoxication and hypercalcemia. More recent work has broadened this view by showing that some CYP24A1-derived metabolites, such as 24,25(OH)_2_D, possess distinct biological activities, especially in skeletal biology and cartilage homeostasis. In this light, CYP24A1 is better conceptualized as a context-dependent determinant of local catabolic flux and signal duration, dynamically shaping the amplitude and persistence of 1,25(OH)_2_D signaling rather than serving merely as a terminal clearance enzyme.^88^^,^^92^^,^^93^

Transcriptional regulation of CYP24A1: CYP24A1 is one of the most strongly inducible targets of the 1,25(OH)_2_D-VDR axis and can be upregulated by several orders of magnitude, forming a classical negative feedback loop. Fibroblast growth factor 23 (FGF23) and PTH further refine this regulation: FGF23 promotes CYP24A1 expression and suppresses CYP27B1, thereby limiting 1,25(OH)_2_D synthesis, whereas PTH exerts the opposite effect by stimulating CYP27B1 and inhibiting CYP24A1, enhancing 1,25(OH)_2_D production. Together, these endocrine signals establish a finely tuned balance among PTH, FGF23, and vitamin D metabolism.^94^^,^^95^^,^^96^

Epigenetically, the methylation status of the CYP24A1 promoter varies substantially across tissues and disease states. In several solid tumors, including prostate, colorectal, and lung cancer, abnormal CYP24A1 promoter methylation is associated with altered gene expression and may influence tumor behavior and responsiveness to vitamin D analogues.^97^^,^^98^^,^^99^ In placental tissue, distinctive CYP24A1 methylation patterns have been linked to local 1,25(OH)_2_D levels and immune tolerance during pregnancy, suggesting that epigenetic programming of this enzyme contributes to tissue-specific microcircuit phenotypes at the maternal-fetal interface.^85^^,^^86^^,^^100^

Post-transcriptionally, microRNA-125b can bind to the 3′ UTR of CYP24A1 and suppress its translation, thereby reducing protein abundance. Dysregulation of this and other microRNAs has been reported in chronic kidney disease, gestational diabetes, and certain malignancies, adding an additional regulatory layer to CYP24A1 expression.^101^^,^^102^^,^^103^^,^^104^ In multiple tumor types, CYP24A1 overexpression leads to accelerated inactivation of 1,25(OH)_2_D, blunting its antiproliferative and pro-apoptotic actions and correlating with tumor progression, treatment resistance, and poor prognosis. Experimental inhibition of CYP24A1 can enhance the antitumor activity of vitamin D and its analogues in preclinical models.^82^^,^^105^^,^^106^^,^^107^^,^^108^^,^^109^

#### Enzyme kinetics and substrate competition

CYP27B1 and CYP24A1 share 25(OH)D as a substrate: CYP27B1 catalyzes its conversion to the active hormone 1,25(OH)_2_D, whereas CYP24A1 hydroxylates both 25(OH)D and 1,25(OH)_2_D to generate a cascade of less active or inactive metabolites. When substrate availability is limited, these enzymes compete for substrate; under such conditions, modest shifts in expression or activity can substantially alter the balance between activation and inactivation. In inflammatory microenvironments, CYP24A1 is robustly induced by the combined actions of 1,25(OH)_2_D and pro-inflammatory cytokines, often gaining a metabolic advantage over CYP27B1 in settings where 25(OH)D is still adequate but inflammation is sustained.^88^^,^^110^^,^^111^ This kinetic imbalance, where catabolism outpaces activation, underpins what we term metabolic vitamin D resistance. Conceptually, this acquired state shares with inherited vitamin D resistance the feature of impaired downstream responsiveness despite substrate availability, but it differs in cause, reversibility, and tissue specificity. It arises when pro-inflammatory cytokines (e.g., TNF-α) simultaneously suppress VDR and upregulate the catabolic enzyme CYP24A1, creating a localized sink that degrades 1,25(OH)_2_D_3_ before effective VDR-mediated signaling can be sustained.

Mathematical modeling suggests that the CYP24A1/CYP27B1 ratio is a key determinant of 1,25(OH)_2_D steady-state levels in both the circulation and tissues. Relative overexpression of CYP24A1 can markedly reduce peak 1,25(OH)_2_D concentrations and shorten the signaling window, whereas insufficient CYP24A1 activity can prolong exposure and increase the risk of hypercalcemia.^33^^,^^86^^,^^93^^,^^94^^,^^112^ Patterns consistent with increased CYP24A1/CYP27B1 ratios have been documented in chronic inflammatory diseases, chronic kidney disease and various solid tumors, where they correlate with disease activity, complication risk, and responsiveness to vitamin D-based interventions.^79^^,^^82^^,^^103^^,^^104^^,^^107^^,^^108^

Overall, CYP24A1 operates as a gatekeeper in two complementary senses. First, through its catalytic activity it constrains excessive 1,25(OH)_2_D signaling and prevents toxic accumulation. Second, via its metabolite profile and potential non-catalytic interactions, it helps set tissue-specific thresholds and shapes the qualitative features of vitamin D responses. When CYP24A1 is excessively activated, microcircuits tend to be shifted toward a low 1,25(OH)_2_D/VDR signaling state. The net biological consequences of such shifts need to be interpreted in light of the full metabolite spectrum and the status of downstream pathways, rather than solely from total 25(OH)D measurements.

### Cell-type-specific microcircuits: The molecular basis of functional specialization

#### Immune cells: Rapid responders and precise modulators

Vitamin D microcircuits in the immune system are notable for their high inducibility and marked cell-type specificity. Monocytes and macrophages are the best-characterized model.

TLR-CYP27B1-antimicrobial peptide axis: PAMPs activate TLRs and markedly upregulate CYP27B1 and VDR expression, rapidly converting 25(OH)D to 1,25(OH)_2_D *in situ*. Via VDR-mediated transcriptional activation, locally generated 1,25(OH)_2_D induces antimicrobial effector programs, including increased expression of CAMP, which encodes the cathelicidin precursor hCAP18 that is subsequently processed to the active peptide LL-37, as well as DEFB4, thereby linking vitamin D signaling to innate antimicrobial defense.^113^^,^^114^^,^^115^ Notably, low 25(OH)D levels blunt the cathelicidin response to TLR stimulation; serum 25(OH)D concentrations of at least ∼30 ng/mL are generally considered necessary to support a reasonably robust antimicrobial peptide response.

Autophagy and inflammasome regulation: 1,25(OH)_2_D upregulates autophagy-related genes, including ATG5, BECN1, and LC3, promotes autophagosome formation and autophagosome-lysosome fusion in monocytes/macrophages, and thereby enhances clearance of intracellular pathogens such as *Mycobacterium tuberculosis*.^116^ Vitamin D signaling also intersects with the NLRP3 inflammasome: enhanced autophagy facilitates removal of damaged mitochondria and reduces ROS-driven NLRP3 activation. Modulation of this autophagy-NLRP3 axis is considered an important mechanism by which vitamin D attenuates chronic inflammation and infection-related tissue injury.^116^^,^^117^

Immune regulation and tolerance induction: 1,25(OH)_2_D drives DCs toward a semi-mature phenotype, with downregulation of CD80/CD86 and MHC class II, reduced secretion of IL-12 and IL-23, and a marked increase in IL-10. The resulting tolerogenic DCs acquire an enhanced capacity to induce regulatory T cells (Treg). Vitamin D signaling suppresses Th1 (IFN-γ) and Th17 (IL-17) responses while promoting the differentiation and functional maintenance of Foxp3^+^ Treg, shifting the balance from a pro-inflammatory state toward immune regulation and tolerance. This DC-T-cell microcircuit is considered a key molecular pathway through which vitamin D contributes to the pathogenesis and remission of autoimmune diseases such as multiple sclerosis and inflammatory bowel disease.^118^^,^^119^

From a microcircuit perspective, immune cells function as “rapid-response organs” for vitamin D, whereas the kidney and bone act as slower, long-term regulators of systemic mineral balance.

#### Epithelial barrier systems: Homeostasis and injury repair

Vitamin D microcircuits in epithelial cells occupy a central position in maintaining barrier integrity, defending against external insults and coordinating tissue repair.

Intestinal epithelium: enterocytes express a near-complete repertoire of vitamin D transport and metabolic components, including brush-border megalin/cubilin-mediated endocytosis of DBP-25(OH)D complexes, CYP27B1-mediated local activation and CYP24A1-mediated degradation. Together, these modules create a highly localized vitamin D microenvironment at the interface between the lumen and the lamina propria.^120^^,^^121^ VDR directly regulates the expression and subcellular distribution of tight junction proteins (claudins, occludin, ZO-1) and adherens junction proteins, thereby maintaining epithelial integrity and selective permeability; VDR deletion or deficiency leads to tight-junction rearrangement, increased epithelial permeability and aggravated mucosal injury in models of inflammatory bowel disease.^33^^,^^120^^,^^122^

Intestinal epithelium-specific *Cyp24a1* deletion enhances local small-intestinal 1,25(OH)_2_D-VDR signaling, increases intestinal calcium absorption and, without causing hypercalcemia, lowers PTH and attenuates secondary hyperparathyroidism in chronic kidney disease models. These findings indicate that intestinal CYP24A1 plays a critical role in “setting” the mucosal vitamin D activity threshold,^33^^,^^123^ although current evidence derives mainly from animal models and limited human tissue studies, and its generalizability requires further prospective data.

Alveolar epithelium: type II alveolar epithelial cells can locally synthesize 1,25(OH)_2_D and modulate surfactant production, tight junction expression and antimicrobial peptide release.^124^ In models of acute lung injury and chronic airway inflammation, an intact vitamin D/VDR axis helps preserve the alveolar epithelial barrier, reduces epithelial-mesenchymal transition (EMT) and limits inflammatory cytokine production and protein leakage; conversely, VDR deficiency leads to marked disruption of tight and adherens junctions, increased permeability and more severe lung injury.^125^ Clinically, vitamin D deficiency has been associated with greater severity and higher exacerbation risk in chronic obstructive pulmonary disease and asthma, supporting an important role for alveolar epithelial vitamin D microcircuits in pulmonary defense and inflammatory repair.^121^^,^^125^

Epidermal keratinocytes: basal and spinous layer keratinocytes are not only the main site of cutaneous vitamin D_3_ synthesis but also express CYP27B1, CYP24A1 and VDR, forming a skin-local vitamin D microcircuit. Local 1,25(OH)_2_D signaling orchestrates keratinocyte proliferation-differentiation programs, keratin and lipid synthesis, and expression of antimicrobial peptides such as cathelicidin and β-defensins, thereby playing a central role in barrier formation and defense against bacterial and viral pathogens. In disorders such as psoriasis and atopic dermatitis, disruption of this microcircuit—through downregulation of VDR, upregulation of CYP24A1 or abnormal ultraviolet exposure—is closely linked to epidermal hyperproliferation, barrier breakdown and chronic inflammation, and provides a mechanistic rationale for topical vitamin D analogues.^126^^,^^127^^,^^128^

#### Central nervous system: Neuro-glial networks

Neurons: neurons in several brain regions, including the hippocampus, prefrontal cortex, and substantia nigra, express VDR and selected vitamin D-metabolizing enzymes, with higher expression during development and persistent, albeit lower, levels in adulthood. 1,25(OH)_2_D upregulates neurotrophic factors such as BDNF, NGF, and GDNF, modulates calcium channels and genes involved in dopamine synthesis, and participates in synaptic plasticity, neurogenesis, and antioxidant defense.^87^^,^^129^ In models of Parkinson’s disease and Alzheimer’s disease, appropriate vitamin D supplementation or VDR agonists reduce neuronal apoptosis, lessen α-synuclein or β-amyloid aggregation and improve cognitive or motor behavioral outcomes.^130^^,^^131^

Microglia: as the resident immune cells of the central nervous system, microglia express CYP27B1 and high levels of VDR, enabling focal production of 1,25(OH)_2_D in response to pathological stimuli and modulation of their own activation states through VDR signaling.^88^^,^^132^ In models of multiple sclerosis and stroke, vitamin D signaling tends to suppress “classically” activated M1-like pro-inflammatory microglia (characterized by high IL-1β, TNF-α, and iNOS expression) while promoting a shift toward M2-like anti-inflammatory/repair phenotypes, thereby reducing demyelination, neuronal injury, and glial scar formation. In parallel, vitamin D can modulate the NLRP3 inflammasome, NF-κB, and oxidative stress pathways, dampening chronic microglia-mediated neuroinflammation.^89^^,^^132^^,^^133^

Astrocytes and blood-brain barrier (BBB) microcircuits: pathological and confocal immunofluorescence studies in Parkinson’s disease have identified a subset of CYP27B1-positive reactive astrocytes in affected regions such as the substantia nigra and frontal cortex. These cells are virtually absent in control brains but are markedly enriched in PD tissue and can phagocytose and degrade α-synuclein aggregates; they are frequently juxtaposed to Lewy body-negative dopaminergic neurons, suggesting that this astrocytic subpopulation may contribute to neuroprotection by locally activating vitamin D pathways.^89^^,^^134^

BBB microcircuits: At the level of the BBB, brain microvascular endothelial cells and ependymal/choroid plexus epithelial cells express VDR and, in some settings, endocytic receptors such as megalin, which can mediate transendothelial transport and clearance of vitamin D-binding protein-vitamin D metabolite complexes. Through VDR-dependent mechanisms, 1,25(OH)_2_D strengthens endothelial tight junctions and mitigates ischemia-reperfusion-induced BBB disruption and cerebral edema, whereas functional alterations in megalin and related receptors may affect the cerebral utilization of vitamin D metabolites and the neuroinflammatory milieu.^54^^,^^135^ Thus, astrocytes, endothelial cells and ependymal cells together form a multicellular cooperative vitamin D microcircuit that is critical for maintaining BBB integrity and immune homeostasis within the brain.

### Pathophysiology: Microcircuit reprogramming by inflammation and aging

Observational studies have repeatedly linked low vitamin D status to an increased risk of cognitive decline.^5^ Meta-analyses suggest that individuals in the lowest 25(OH)D quantile have a higher risk of dementia than those in the highest quantile, with each 10 nmol/L increase in 25(OH)D associated with a modest reduction in risk.^136^ These associations do not in themselves prove causality, but they illustrate how systemic vitamin D status and tissue-level microcircuits may converge on brain outcomes and motivate a closer examination of the underlying pathophysiology.

#### Inflammation-induced metabolic reprogramming

A key conceptual issue is how vitamin D-immune signaling relates to metabolic vitamin D resistance. In our view, these are not competing frameworks but potentially sequential states within the same microcircuit. During acute innate immune activation, TLR-driven induction of CYP27B1 and VDR supports local production of 1,25(OH)_2_D and downstream antimicrobial and immunoregulatory programs.^22^^,^^115^ By contrast, when inflammatory signaling becomes sustained, cytokine- and stress-responsive pathways such as NF-κB and JAK-STAT increasingly favor CYP24A1 induction, distort the CYP27B1/CYP24A1 balance, and reduce effective VDR-dependent responsiveness.^137^^,^^138^ The result is a transition from adaptive local activation to a catabolism-dominant, low-response state in which adequate circulating 25(OH)D no longer guarantees adequate tissue signaling.

Redox coupling at the mitochondrial interface: CYP27B1 is a mitochondrial type I cytochrome P450 and requires NADPH-derived reducing equivalents delivered via the mitochondrial electron-transfer chain. This requirement makes local 1,25(OH)_2_D generation inherently sensitive to redox balance: oxidative stress and inflammatory metabolic rewiring can divert NADPH and limit effective electron flux even when 25(OH)D supply is sufficient.^1^^,^^2^^,^^14^ In parallel, inflammatory programmes that accompany redox imbalance (NF-κB; IL-6-STAT3) frequently induce CYP24A1 and may reduce VDR abundance or responsiveness, accelerating catabolism and shortening the signaling window.^15^^,^^86^^,^^137^ Together, these shifts provide a biochemical rationale for the frequent decoupling of circulating 25(OH)D from tissue-level exposure-response and for context-dependent non-response to supplementation.

NF-κB-CYP24A1 axis: multiple pro-inflammatory cytokines—including TNF-α, IL-1β, and IL-6—induce CYP24A1 expression via NF-κB signaling. This accelerates local degradation of 25(OH)D and 1,25(OH)_2_D and can create an “inflammatory vitamin D resistance/exhaustion” state. Consistent patterns of CYP24A1 upregulation with enhanced catabolism have been documented in experimental colitis, renal tubular epithelial cells, and several tumor cell lines.^123^^,^^139^ In parallel, 1,25(OH)_2_D itself exerts context-dependent bidirectional regulation on CYP24A1: in some settings, it reinforces catabolic inactivation, whereas in others it cooperates with VDR and co-regulators to enhance transcription of specific target genes.^86^^,^^138^^,^^140^

JAK-STAT crosstalk: IL-6 can downregulate CYP27B1 and upregulate CYP24A1 via JAK-STAT3 signaling, weakening local vitamin D-mediated anti-inflammatory responses in diseases such as inflammatory bowel disease.^137^ Chronic activation of the IL-6/STAT3/SOCS3 axis in ulcerative colitis and colitis-associated cancer has been linked to epithelial survival, persistent inflammation and tumorigenesis, and is accompanied by a shift in the CYP27B1/CYP24A1 balance toward increased catabolism.^35^^,^^138^^,^^141^^,^^142^^,^^143^^,^^144^ At present, this metabolic reprogramming pattern is supported mainly by evidence from IBD, murine colitis and selected tumor models; whether similar patterns operate across other organs and chronic inflammatory conditions remains to be systematically evaluated.

Epigenetic reprogramming: chronic inflammation not only alters immediate transcriptional responses but can also stabilize a state of reduced vitamin D responsiveness through epigenetic remodeling. In human cohorts with infections, autoimmune diseases and cancer, altered DNA methylation and histone modifications in promoter regions of VDR and key vitamin D-metabolizing enzymes (CYP27B1, CYP24A1) have been associated with decreased gene expression and greater disease activity.^145^^,^^146^ Abnormal methylation and histone modification of regulatory proteins further contribute to misaligned expression profiles, suggesting that inflammatory microenvironments can durably reshape vitamin D metabolic pathways through epigenetic remodeling.^35^^,^^99^^,^^147^

In dextran sulfate sodium-induced colitis models, class I histone deacetylase (HDAC) inhibitors restore histone H_3_ acetylation, enhance VDR-dependent transcriptional activity and ameliorate inflammation and epithelial barrier disruption. These protective effects are markedly attenuated in Vdr−/− mice or under vitamin D deficiency, implying that HDAC inhibition acts, at least in part, by reversing epigenetic suppression of VDR signaling.^148^^,^^149^^,^^150^ Collectively, these data indicate that HDAC-mediated deacetylation and related chromatin changes constitute a potentially reversible node within the inflammation-barrier dysfunction-vitamin D axis.

#### Ageing-related systemic changes

Aging is accompanied by multi-level functional decline of the vitamin D system, and these changes interact with “inflammatory microcircuits.”

Declining renal function: in older individuals, renal 1α-hydroxylase (CYP27B1) activity and 1,25(OH)_2_D production capacity decline with age, in parallel with reductions in glomerular filtration rate and tubular mass.^151^^,^^152^ In addition, the kidney’s responsiveness to PTH and FGF23-Klotho signaling is attenuated, further limiting the aging kidney’s capacity to generate active vitamin D.^153^^,^^154^

Reduced megalin/cubilin expression: aging is associated with decreased expression of megalin and cubilin in the renal cortex, which impairs the proximal tubular reabsorption of filtered DBP-25(OH)D complexes and reduces substrate supply for intrarenal vitamin D metabolism.^28^^,^^61^ In aged rats, both the abundance and membrane localization of megalin and cubilin are altered, coinciding with reduced protein reabsorption capacity.^155^ These changes may contribute to tissue-level vitamin D insufficiency even when serum 25(OH)D appears adequate.

Chronic low-grade inflammation: vitamin D deficiency and “inflammaging” (chronic low-grade inflammation of aging) appear to reinforce each other. Older adults with lower 25(OH)D concentrations tend to have higher levels of CRP, IL-6, and other inflammatory markers, and MR analyses suggest a potential causal component in the association between 25(OH)D and systemic inflammation.^156^^,^^157^^,^^158^ At the tissue level, persistent inflammatory signaling can upregulate CYP24A1 while dampening CYP27B1 and VDR, whereas reduced vitamin D signaling impairs anti-inflammatory responses and clearance of senescent cells. This bidirectional interaction favors the establishment of pro-inflammatory microcircuits and creates a vicious cycle: inflammaging and metabolic stress → NF-κB/IL-6–STAT3 activation → CYP24A1 upregulation with reduced CYP27B1/VDR signaling → diminished anti-inflammatory and senolytic capacity → further escalation of inflammation.

Altered VDR function: in several ageing-related target tissues—such as skeletal muscle, kidney, parathyroid gland, and subsets of immune cells—VDR expression and vitamin D-metabolizing enzymes show age-related changes. Epigenetic alterations in the VDR promoter region, including increased DNA methylation and histone deacetylation, together with shifts in the composition of co-activator and co-repressor complexes, can reduce the ability of VDR to recruit transcriptional machinery.^83^^,^^157^^,^^158^ As a result, the same circulating 1,25(OH)_2_D concentration may elicit a blunted gene expression response in older individuals compared with younger adults.

**Figure 2:**
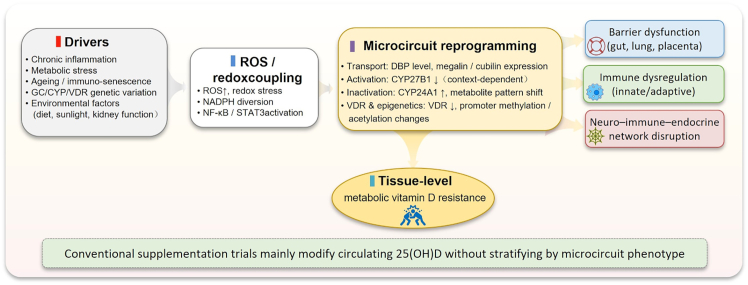
Redox-sensitive reprogramming of vitamin D microcircuits across tissues Systemic and local drivers (e.g., inflammation, aging, chronic disease states, and genetic variation) shape tissue vitamin D handling through coordinated changes in transport and metabolism. A central ROS/redox node links upstream drivers to microcircuit remodeling: (1) mitochondrial redox metabolism and NADPH-dependent electron transfer constrain CYP27B1-mediated local activation of 25(OH)D; and (2) ROS-inflammatory signaling (e.g., NF-κB/STAT3) promotes CYP24A1 induction and may attenuate VDR signaling, collectively shortening the effective signaling window and contributing to context-dependent “metabolic vitamin D resistance.” The net result is potential uncoupling between circulating 25(OH)D supply and tissue-level 1,25(OH)_2_D exposure and transcriptional output. Created by the authors; not adapted from previously published material.

Taken together, these lines of evidence suggest that chronic inflammation, metabolic stress, aging, and GC/CYP/VDR genetic variation do not operate as isolated factors. Rather, they converge at the cellular level to reshape vitamin D microcircuits through recurrent patterns of CYP27B1 downregulation, CYP24A1 upregulation, and altered VDR expression and epigenetic control. The resulting tissue-specific phenotypes—characterized by decoupling of serum 25(OH)D from local 1,25(OH)_2_D signaling and a shift toward low-responsiveness states—manifest clinically as barrier dysfunction, immune imbalance, and disruption of neuro-immune-endocrine loops, and can be conceptualized as forms of “metabolic vitamin D resistance” (Figure 2).

### Genetic variation and personalized medicine

#### GC polymorphisms

The GC gene encodes DBP and represents the single most important genetic locus influencing circulating 25(OH)D concentrations. Common GC variants account for a substantial proportion of SNP-explained variance in 25(OH)D and constitute one of the strongest effect loci identified to date.^159^^,^^160^

Marked ancestral differences exist in GC allele frequencies. GC1f-related alleles and specific rs7041/rs4588 haplotype combinations are much more prevalent in individuals of African ancestry than in those of European or East Asian origin. These genetic patterns contribute to systematically lower total 25(OH)D concentrations in African-ancestry populations, yet estimates of free or bioavailable 25(OH)D are often comparable to those in White populations.^50^^,^^161^^,^^162^ This provides both genetic and physiological support for the concept of race- or ancestry-specific reference ranges for vitamin D status.

MR studies using genetic instruments constructed from GC, CYP2R1, DHCR7, and CYP24A1 loci have provided relatively robust causal inference for certain outcomes, particularly skeletal endpoints, while yielding largely null or inconsistent evidence for many non-skeletal diseases.^163^^,^^164^^,^^165^ These findings highlight both the strengths and the limitations of MR as applied to vitamin D. On the one hand, GC- and CYP-based instruments capture lifelong, genetically anchored differences in 25(OH)D, thereby avoiding many of the confounding and reverse-causation issues that plague observational studies. On the other hand, the effect sizes encoded by common variants are modest and correspond to small, chronic exposure differences; moreover, MR instruments reflect overall circulating 25(OH)D levels and cannot resolve tissue-specific exposure within distinct vitamin D microcircuits across organs.^166^^,^^167^

#### CYP enzyme gene variants

CYP27B1 and CYP24A1 occupy bottleneck positions in the activation and inactivation pathways of vitamin D metabolism. Rare loss-of-function variants in these genes often give rise to striking monogenic phenotypes, whereas common variants exert only modest effects on 25(OH)D concentrations in the general population.

CYP27B1 deficiency: biallelic loss-of-function mutations in CYP27B1 cause vitamin D-dependent rickets type 1 A (VDDR1A). Affected infants typically present with refractory hypocalcemia, secondary hyperparathyroidism, and severe rickets, along with very low 1,25(OH)_2_D despite sufficient or elevated 25(OH)D.^168^^,^^169^ Heterozygous carriers usually have no overt symptoms, but under conditions of low calcium/vitamin D intake or coexisting disturbances of mineral metabolism they may exhibit mild biochemical abnormalities or subclinical skeletal changes.^170^

CYP24A1 deficiency: pathogenic variants in CYP24A1 underlie idiopathic infantile hypercalcemia, characterized clinically by early-onset hypercalcemia, hypercalciuria, and nephrocalcinosis, and, in severe cases, by impaired renal function.^33^^,^^171^ In adults, CYP24A1 mutations contribute to increased susceptibility to nephrolithiasis.^172^ Functional studies have shown that different variants often only partially reduce enzyme activity *in vitro*, consistent with the wide phenotypic spectrum observed in carriers—from asymptomatic individuals to patients with recurrent kidney stones.

These rare, high-effect variants underscore the continuum between “monogenic endpoints” and “common variation” in the vitamin D metabolic pathway. At one extreme, classical VDDR1A and idiopathic infantile hypercalcemia exemplify severe disruption of activation or inactivation, respectively; at the other, numerous common, low-effect SNPs collectively shape inter-individual variation in 25(OH)D levels and disease risk. This continuum provides a genetic rationale for exploring targeted interventions in high-risk families and phenotypic extremes, while recognizing that in the general population most variation reflects the additive effects of many small genetic and environmental influences.

#### VDR variants

The VDR gene, located on 12q13.11, harbors more than 200 described common polymorphisms. Among these, four variants—FokI (rs2228570), BsmI (rs1544410), ApaI (rs7975232), and TaqI (rs731236)—have been most extensively studied.

FokI (rs2228570): the C/T substitution at the translation initiation site generates two protein isoforms: the F allele encodes a VDR protein that is three amino acids shorter than the f allele product, with higher transcriptional activity in most experimental systems. Epidemiological studies have reported associations between FokI genotype and bone mineral density, fracture risk and responsiveness to vitamin D and calcium supplementation, with the F allele often linked to more favorable skeletal outcomes or a more pronounced response to supplementation, especially among postmenopausal women and individuals with low baseline intake.^173^^,^^174^ Nevertheless, effect sizes are generally small to moderate.

BsmI-ApaI-TaqI 3′ haplotypes: the BsmI, ApaI, and TaqI polymorphisms in the 3′ region of VDR are in strong linkage disequilibrium and form several common haplotypes that influence mRNA stability and expression levels.^175^^,^^176^ Certain haplotypes are associated with lower VDR expression, lower baseline 25(OH)D concentrations, and blunted increases in 25(OH)D after oral supplementation or UV exposure.^175^ In one cohort, carriers of specific BsmI-ApaI-TaqI haplotypes had the smallest rise in 25(OH)D and the highest proportion of “non-responders” after 12 weeks of supplementation and sunlight exposure, supporting a link between this haplotype block and poor 25(OH)D increment and supplementation responsiveness.^177^

VDR genotypes and heterogeneity of response: FokI, BsmI, ApaI, TaqI, and their haplotypes can explain part of the between-individual heterogeneity in bone mineral density, PTH dynamics, and 25(OH)D increments observed in supplementation trials, but the contribution of any single locus is limited and typically accounts for only a small fraction of the total variance.^178^ Rather than acting as simple “on/off” switches, these genetic variants appear to pre-set organ-specific “gain factors” for vitamin D signaling—modulating how strongly different tissues respond to a given circulating 25(OH)D level—rather than merely determining overall vitamin D concentrations.

### Clinical translation: From mechanisms to precision intervention

#### Reappraising biomarkers

Although total 25(OH)D remains the most practical first-line indicator of vitamin D status in current clinical practice,^179^ from a microcircuit perspective it is essentially an upstream supply marker. It captures overall systemic availability but is poorly suited to describing tissue-specific vitamin D exposure or microcircuit phenotypes. Drawing on existing evidence, we have therefore summarized candidate markers most likely to enter clinical use or trial design in the near term and organized them into a framework spanning systemic exposure, bone-kidney feedback, and the inflammatory milieu (Table 1).

**Table 1:** Core candidate markers of the vitamin D cellular endocrine system: evidence strength and key knowledge gaps

Pathway component	Marker (abbreviation)	Key evidence types	Evidence strength	Measurement and availability	Major unresolved questions
Transport/systemic exposure	total 25(OH)D	large cross-sectional and prospective cohorts; RCTs on bone density/fracture; multi-disease MR	strong (skeletal outcomes)/moderate (most non-skeletal outcomes)	LC-MS/MS or immunoassay widely standardized; currently the only routinely comparable exposure marker	what is the optimal threshold and “upper limit of effect” for non-skeletal outcomes? Should thresholds be stratified by population, disease, or age group?
Transport/systemic exposure	free/bioavailable 25(OH)D	*in vitro* binding studies; small patient cohorts (CKD, cardiovascular, immune diseases); correlations with bone density/muscle strength	moderate	requires DBP/albumin assays or specialized platforms; limited standardization	in states with altered albumin or DBP (CKD, pregnancy), does free/bioavailable 25(OH)D outperform total 25(OH)D in predicting outcomes or guiding supplementation? Is the incremental value sufficient to alter clinical decision-making?
Transport/exposure+ inactivation	VMR: 24,25(OH)_2_D/25(OH)D	metabolomics; bone density/fracture cohorts; high-dose supplementation trials	moderate	requires LC-MS/MS; feasible but not routine; no consensus cut-off	can VMR reliably reflect individual metabolic flux under different dosing regimens? Could it serve as a tool for assessing safety and “overdose” risk, and provide incremental information for non-skeletal outcomes?
Transport/systemic exposure	serum DBP concentration + GC genotype	population cohorts; associations between GC polymorphisms and 25(OH)D levels/responses; limited functional evidence	moderate	DBP ELISA or mass spectrometry feasible but not routine; GC genotyping common in research	what is the quantitative contribution of GC haplotypes and DBP isoforms to substrate availability and supplementation response? Could simple algorithms “genotype-correct” total 25(OH)D to improve risk stratification?
Activation (CYP27B1)	serum 1,25(OH)_2_D	CKD and calcium-phosphate metabolism studies; limited supplementation trials	moderate (bone-renal axis)/weak (microcircuit level)	technically demanding, costly, sensitive to sample handling; used in few centers	to what extent does serum 1,25(OH)_2_D reflect extra-renal microcircuit activity? In severe deficiency, CKD, or genetic defects, are there clearer applications (monitoring active analog therapy)?
Receptor feedback/downstream function	PTH	Established clinical test; extensive bone-renal axis studies; some cardiovascular/mortality cohorts	strong (bone-renal axis)/moderate (other outcomes)	widely available, moderate cost; guidelines already recommend combined 25(OH)D + PTH assessment	what is the quantitative relationship between PTH and “true vitamin D activity” at the microcircuit level? Should PTH be routinely included as a secondary endpoint in non-skeletal trials?
Receptor feedback/downstream function	FGF23	large CKD, cardiovascular, mortality cohorts; mechanistic studies	strong (CKD-bone-cardiovascular axis)/moderate (general population)	feasible but costly; mainly research and specialized centers	in the general population, is there a stable link between FGF23 and vitamin D microcircuit status? Should FGF23 be incorporated into high-dose supplementation trials as part of the safety/cardiovascular risk framework?
Inflammation-microcircuit crosstalk	CRP, IL-6	numerous observational studies (inverse correlation with 25(OH)D); some MR suggesting causality; inconsistent supplementation effects	moderate (association)/weak (specificity)	routine clinical assays, widely accessible	how much explanatory power do non-specific markers (CRP, IL-6) have in the “inflammation-vitamin D axis”? How should they be combined with more specific metabolic/receptor markers to avoid misinterpretation?
Integrated microcircuit phenotype	PBMC VDR expression or simplified VDR target gene panel (“vitamin D response score prototype”)	*in vitro* 1,25(OH)_2_D stimulation + transcriptomics; small patient PBMC studies	weak-moderate	requires PBMC isolation and RT-qPCR/flow cytometry; feasible but not standardized	can a small set of genes/proteins be distilled into a reproducible “vitamin D response score” usable in large cohorts and RCTs? What is its incremental predictive value and reproducibility?

Note: ∗ Key evidence types refer to a generalized synthesis based on *in vitro* experiments, animal models, human observational studies, and intervention trials; they do not represent a formal GRADE assessment. † Evidence strength is expressed as a qualitative ranking (strong/moderate/weak) provided for ease of reading. It highlights the relative weight within the current knowledge landscape rather than the appraisal of any single study. VMR, vitamin D metabolite ratio; PTH, parathyroid hormone.

Among the core exposure markers, free and bioavailable 25(OH)D represent an extension of the “free hormone hypothesis.” They are defined as the sum of the free and albumin-bound fractions and have shown incremental predictive value beyond total 25(OH)D in several cardiovascular, chronic kidney disease, and pregnancy cohorts.^180^^,^^181^ However, these gains have not been consistent across studies, and current assays are technically demanding and lack full standardization. For large-scale epidemiology and routine clinical work, total 25(OH)D therefore remains the preferred exposure marker,^182^ whereas free/bioavailable 25(OH)D may be reserved for more focused mechanistic questions or prespecified subgroup analyses.^70^^,^^180^^,^^183^

Beyond exposure, functional readouts of microcircuit activity are equally important. Table 1 lists downstream markers with translational potential, including PTH, FGF23, CRP/IL-6, and simplified PBMC-based VDR-related readouts, which together capture bone-kidney feedback, inflammatory tone, and selected aspects of microcircuit responsiveness. At present, these biomarkers are mainly used as exploratory or secondary outcomes in cohort studies and trials; insufficient evidence supports their use as primary status indicators at the guideline level. Nevertheless, they offer a promising toolkit for delineating tissue-specific phenotypes of “functional vitamin D insufficiency” or “metabolic vitamin D resistance,” as outlined in Table S1.^184^^,^^185^^,^^186^^,^^187^^,^^188^^,^^189^^,^^190^^,^^191^^,^^192^

As highlighted in the multiscale overview in Figure 1, the majority of currently accessible markers cluster at the circulatory level, while direct indicators of tissue and microcircuit status remain mostly at the experimental stage. This imbalance helps explain why most previous trials have relied almost exclusively on total 25(OH)D and why they have struggled to capture heterogeneity across organs and disease contexts.

#### Toward personalized supplementation strategies

Standard-dose vitamin D supplementation clearly benefits skeletal endpoints and remains the backbone of current guideline recommendations.^5^^,^^9^ For non-skeletal outcomes—including cancer, cardiovascular events, depressive disorders, and infections—large randomized trials and MR studies have generally reported null or small effects, with possible benefit confined to particular subgroups or contexts.^100^^,^^179^ These results argue against a single “magic threshold” and suggest that baseline exposure, genotype, inflammatory status, and tissue-specific microcircuits all need to be considered when deciding who might benefit, and how.

Genotype-informed dosing: GC (DBP) variants rs7041 and rs4588 and their haplotypes (including DBP2/GC2 alleles) exert appreciable effects on baseline 25(OH)D concentrations and on the increment achieved with a given oral dose.^193^^,^^194^^,^^195^^,^^196^^,^^197^ VDR polymorphisms (FokI, BsmI-ApaI-TaqI haplotypes) and CYP27B1/CYP24A1 variants further modulate how strongly tissues respond to circulating vitamin D and to supplementation. In principle, this information could be used to flag individuals who are more likely to remain at the low end of the biological response curve unless given higher or longer dosing, or alternative formulations (Table 1). At present, however, these genotypes are better viewed as modifiers of dose-response than as stand-alone indications for treatment, and robust trials that explicitly test “genotype-guided” dosing strategies are still lacking.^198^^,^^199^^,^^200^

Inflammatory status: observational data indicate an inverse association between 25(OH)D and CRP, and MR analyses suggest a potential causal association between lower 25(OH)D and higher systemic inflammation.^201^^,^^202^ In intervention studies, effects of vitamin D on hs-CRP and IL-6 are modest and variable, with some suggestion that people starting with high inflammatory markers may have greater relative reductions.^100^^,^^203^^,^^204^^,^^205^ Mechanistically, pronounced inflammation can shift microcircuits toward a state of functional vitamin D resistance via CYP24A1 upregulation and downregulation of CYP27B1/VDR.^34^^,^^206^ Under such conditions, simply doubling the maintenance dose is unlikely to be a universal solution. This argues for formally testing dose, dosing frequency and co-interventions with anti-inflammatory agents in high-inflammation populations, rather than assuming that a single regimen is appropriate across the board.

Tissue-targeted delivery: strategies to enhance tissue-specific delivery or action are beginning to emerge. These include: (1) CYP24A1 inhibitors, which prolong the half-life and activity of endogenous or exogenous 1,25(OH)_2_D and show synergistic antitumor or immunomodulatory effects in preclinical models^207^^,^^208^^,^^209^; (2) prodrugs or analogues with modified affinity for megalin/cubilin or altered lipophilicity, designed to favor uptake at intestinal, renal or barrier sites^27^; and (3) nanoparticle- or liposome-based formulations aimed at macrophages, tumor tissues, or the central nervous system.^27^^,^^210^^,^^211^ For now, these approaches mostly sit in the preclinical or early translational arena and are readout using mechanistic biomarkers, as summarized in Table S1. Their relevance for routine supplementation or population-level prevention will depend on whether they can be translated into safer, more effective and cost-conscious regimens in defined high-risk groups.

In practice, these considerations point away from a single numeric threshold and toward decisions that start with total 25(OH)D but are tempered by ancestry, GC/CYP/VDR genotype, inflammatory state and—where available—simple functional readouts of microcircuit activity.

#### Optimizing clinical trial design

The microcircuit framework also has implications for how vitamin D trials are planned and interpreted.

Stratification and randomization factors: beyond conventional stratification by baseline 25(OH)D, future trials could incorporate GC/DBP genotypes or DBP phenotypes (DBP2 carriers vs*.* non-carriers), baseline inflammatory markers (CRP, IL-6) and simple proxies of barrier or bone-kidney microcircuit status as stratification or minimization factors.^194^^,^^195^^,^^198^ In specific settings—such as chronic kidney disease, inflammatory bowel disease or pregnancy—markers related to megalin-mediated tubular reabsorption or placental transfer could be added as exploratory outcomes to characterize better the functional state of the megalin-DBP-25(OH)D reclamation pathway.^201^^,^^202^^,^^212^^,^^213^

Dynamic monitoring and PK-PD modeling: rather than relying on a single baseline and end-of-trial measurement, serial assessment of total 25(OH)D, free/bioavailable 25(OH)D, PTH, FGF23 and one or two functional readouts (for example, LL-37 or bone turnover markers) would allow simple pharmacokinetic-pharmacodynamic (PK-PD) models to be fitted. These models can show how participants with different genotypes, inflammatory profiles, or microcircuit phenotypes traverse the exposure-response curve under the same dose.^184^^,^^187^^,^^190^^,^^192^ This, in turn, can help distinguish true biological non-responders from under-dosed participants and clarify whether a null clinical result reflects inadequate biological signal or genuinely absent benefit.

Combination interventions: combination strategies also warrant more systematic evaluation. One example is vitamin D plus anti-inflammatory therapy: in IBD, rheumatic diseases and other immune-mediated conditions, small proof-of-concept trials could compare standard immunosuppression alone with regimens that include carefully titrated vitamin D, using microcircuit-sensitive markers as intermediate endpoints.^100^^,^^214^ A second example is vitamin D plus probiotics. Bidirectional interactions between vitamin D status and the gut microbiota have been reported, and several small trials have examined combined vitamin D-probiotic regimens in conditions such as colitis and irritable bowel syndrome, with mixed and largely exploratory results so far.^215^^,^^216^^,^^217^^,^^218^ A third example is vitamin D plus magnesium. Magnesium is an essential cofactor in vitamin D metabolism, and low magnesium status has been associated with blunted 25(OH)D increments after supplementation; early evidence suggests that correcting magnesium deficiency improves the proportion of individuals who reach target 25(OH)D levels.^219^^,^^220^ Magnesium co-supplementation addresses a fundamental biochemical bottleneck: both CYP27B1 (activation) and CYP24A1 (catabolism) are P450 enzymes that obligately require magnesium for electron transfer. Consequently, hypomagnesemia structurally impairs the enzymatic machinery of the vitamin D microcircuit. Correcting magnesium deficits is therefore a prerequisite for restoring catalytic efficiency, particularly in patients unresponsive to high-dose vitamin D monotherapy.

Taken together, more deliberate use of microcircuit-informed stratification, dynamic biomarker panels and rational combinations may help explain why previous trials have yielded heterogeneous results and provide a clearer path toward mechanism-guided, context-aware vitamin D interventions in clinical and public health practice.

### Future directions and research opportunities

#### Technological frontiers

A major next step is to move from bulk averages to cell-resolved maps of vitamin D microcircuits. Single-cell multi-omics—integrating scRNA-seq, scATAC-seq, and spatial transcriptomics—now allows the coordinated expression of VDR, CYP27B1, CYP24A1, and transport components to be charted at cell-type resolution across tissues and disease states.^77^^,^^221^^,^^222^ These approaches can identify microcircuit “hubs” and “bottlenecks” where vitamin D signaling is amplified, buffered, or diverted, and can track how inflammation, fibrosis, or tumor evolution rewire these nodes. Recent spatial transcriptomic studies of developing human retina, for example, have begun to treat “vitamin D metabolic” genes as a defined lipid-soluble vitamin module with marked spatiotemporal dynamics, illustrating how such maps can be constructed in other organs.^223^

In parallel, emerging chemical biology tools are beginning to bring VDR biology into the realm of live-cell imaging. Turn-on fluorescent probes based on conjugate addition-cyclization (TCC) chemistry now enable covalent labeling and visualization of VDR in living cells, allowing real-time monitoring of receptor localization, turnover, and protein-protein interactions.^224^ Coupled with fluorescent vitamin D analogues and advanced microscopy, these tools raise the prospect of directly observing how 1,25(OH)_2_D engages its receptor, co-regulators, and chromatin targets in intact tissue.

Functional genomics provides a complementary path to systematically dissect microcircuit control. CRISPR/Cas9 editing has already implicated SDR42E1 as a key determinant of vitamin D uptake and cancer cell survival, linking a previously obscure gene to both vitamin D homeostasis and tumor biology.^225^ Extending this strategy to pooled, genome-scale CRISPR screens—using transcriptional readouts or vitamin D-responsive reporters—could reveal positive and negative regulators of vitamin D signaling across epithelial, immune, and stromal compartments, generating a prioritized list of druggable nodes within microcircuits.

Finally, artificial intelligence is well placed to integrate heterogeneous data streams into clinically usable risk tools. Machine-learning models such as random forests, XGBoost, and ensemble methods already predict vitamin D deficiency using only questionnaire data, anthropometry, and routine laboratory measures, often with excellent discrimination.^226^^,^^227^^,^^228^ These same frameworks could be expanded to incorporate genotype, metabolomics, inflammatory markers, and longitudinal 25(OH)D trajectories, in order to estimate individual vitamin D requirements, likely responses to supplementation, and risk of adverse outcomes—providing a methodological foundation for AI-assisted dosing decisions.

#### Broadening the conceptual framework

At the biochemical level, there is growing recognition that a single metabolite is unlikely to capture the complexity of vitamin D biology. LC-MS/MS platforms can now quantify panels of vitamin D metabolites from the same sample, enabling calculation of composite indices such as the vitamin D metabolite ratio (VMR; 24,25(OH)_2_D/25(OH)D).^179^^,^^229^^,^^230^ These “vitamin D metabolomes” offer a more dynamic view of synthesis, activation, and catabolism, and may help explain non-linear responses seen in high-dose trials—for example, the paradoxical reduction in bone mineral density observed with chronic 10,000 IU/day supplementation in otherwise vitamin D-replete adults.^179^^,^^231^

Crucially, vitamin D microcircuits do not operate in isolation within single organs. Endocrine signals such as FGF23 and PTH knit intestinal, skeletal, and renal microcircuits into a tightly coupled Ca-P-vitamin D axis.^179^^,^^232^ In addition, extracellular vesicles and exosomes released from the gut, immune system, and tumor microenvironment are increasingly recognized as vehicles that can carry proteins, lipids, and nucleic acids related to vitamin D synthesis and signaling.^233^^,^^234^ Placing vitamin D microcircuits within this broader “inter-organ communication” network may clarify their roles in chronic inflammation, cancer cachexia, and age-related decline.

An evolutionary perspective also enriches the framework. Comparative genomics and functional studies suggest that VDR and vitamin D pathways have undergone a progressive expansion of roles—from ancient functions related to detoxification and oxidative stress management, to modern roles in bone mineralization, immunity, and barrier regulation in vertebrates.^235^^,^^236^ Considering evolutionary constraints (such as skin pigmentation, historical UV exposure patterns, and dietary shifts) may help explain inter-individual variability in vitamin D requirements and the apparent sensitivity of certain “modern” conditions—obesity, immune dysregulation, and mood disorders—to vitamin D status.

#### Translational challenges

Translating a microcircuit-based view of vitamin D into practice will require progress on several practical fronts. First, standardization remains fundamental. Recent international consensus documents reaffirm total serum 25(OH)D as the most robust and comparable marker for assessing vitamin D status at present, while also underscoring the need to harmonize immunoassays with LC-MS/MS through vitamin D standardization programs.^179^^,^^237^ Free or bioavailable 25(OH)D may provide useful complementary information in specific contexts—such as advanced kidney or liver disease or marked DBP polymorphism—but assay methods and reference intervals are not yet sufficiently standardized for routine population screening.^179^^,^^232^^,^^238^ Functional biomarkers downstream of 25(OH)D, including PTH, FGF23, and composite indices such as the VMR, hold promise as more direct readouts of tissue-level vitamin D activity, but require systematic evaluation in prospective cohorts and intervention trials.^179^^,^^229^^,^^230^

Second, cost-effectiveness must be considered explicitly. In high-risk settings—such as older adults, individuals with dark skin in high latitudes, or populations with limited dietary intake—vitamin D supplementation to prevent rickets or osteomalacia and reduce fracture risk appears cost-effective.^239^^,^^240^^,^^241^ By contrast, large randomized trials in generally vitamin D-replete adults have not demonstrated clear benefit of widespread screening or chronic high-dose supplementation for skeletal outcomes, and in some cases have suggested potential harm at very high doses.^231^^,^^237^ Future health-economic modeling should therefore compare simple threshold-based strategies with more nuanced, precision approaches that incorporate genotype, inflammatory status, and microcircuit phenotypes, framing the question as “how to supplement, in whom, and to what target” rather than as a binary “supplement or not.”

Third, there is a regulatory dimension that cannot be overlooked. For mechanism-proximal biomarkers—such as free 25(OH)D, VMR, or tissue-specific microcircuit markers—analytical validity and cross-sectional associations are necessary but insufficient. To influence guideline-level decisions or serve as enrichment criteria, dose-adjustment tools, or companion diagnostics, such biomarkers must pass through formal qualification pathways (e.g., the FDA CDER Biomarker Qualification Program or EMA biomarker qualification), with a clearly defined context of use and prospective validation.^242^^,^^243^ For vitamin D, this means that trialists, regulators, assay manufacturers, and academic consortia will need to collaborate early, embedding microcircuit-relevant endpoints and stratification schemes into trial design rather than attempting to retrofit biomarker claims onto positive or negative clinical endpoints after the fact.

### Conclusions and outlook

The cellular endocrine microcircuit concept shifts our understanding of vitamin D from a single circulating hormone to a distributed, self-regulated network operating across multiple organs. Current evidence indicates that the biological impact of vitamin D is shaped not only by the absolute concentration of serum 25(OH)D, but also by the configuration and dynamic state of local microcircuits—comprising DBP–megalin/cubilin transport, the CYP27B1/CYP24A1 activation-inactivation balance, and VDR-mediated signaling—and by their capacity to respond to inflammatory, metabolic, and aging-related cues.^6^^,^^179^^,^^244^^,^^245^ Circulating 25(OH)D supplies the “fuel”; the architecture and regulation of tissue-specific microcircuits determine how that fuel is used.

Several propositions emerge from this synthesis. First, tissue specificity is fundamental. Differences in the “component mix” and regulatory logic of transporters, metabolic enzymes, and receptors across cell types mean that identical circulating vitamin D levels can translate into very different local exposures and response profiles.^33^^,^^40^^,^^246^ This helps to explain why the same supplementation regimen can yield divergent effects across organs and clinical phenotypes.

Second, inflammation and aging appear to be the dominant modifiers of microcircuit behavior. Pro-inflammatory pathways such as NF-κB and STAT3 can induce CYP24A1 and alter VDR expression, creating a state of “metabolic vitamin D resistance” in inflamed tissues.^35^^,^^93^^,^^247^^,^^248^^,^^249^ Age-related declines in renal function, reduced megalin/cubilin-mediated reabsorption, and epigenetic drift further narrow the effective local window for vitamin D action.^6^^,^^179^^,^^250^ In such contexts, raising serum 25(OH)D alone may be insufficient to restore microcircuit function.

Third, genetic background interacts with environmental and clinical factors to shape an individual’s “vitamin D requirement and response spectrum.” Variants in GC, CYP genes, and VDR influence baseline status, metabolite patterns, and downstream transcriptional responses, and may account for some of the heterogeneity observed in supplementation trials.^193^^,^^197^^,^^251^^,^^252^ Incorporating this layer into trial design and risk stratification is a logical next step toward precision vitamin D medicine.

From a clinical and public health perspective, these insights argue against the expectation that a single threshold or fixed dose can adequately capture the complexity of “vitamin D intervention.” A more realistic agenda will likely involve three levels. At the population level, repeated 25(OH)D measurements, expanded vitamin D metabolite profiling, and joint assessment with inflammation and kidney function could better characterize exposure-response relationships over time. At the organ and cellular levels, single-cell and spatial omics can map microcircuits in key tissues such as gut, skeletal muscle, kidney, and brain, identifying vulnerable nodes that could be targeted pharmacologically. At the intervention level, microcircuit features could be used to refine eligibility criteria, dosing schemes, formulations, and co-interventions in trials, with a particular focus on high-risk, high-yield groups.^6^^,^^7^^,^^179^^,^^250^^,^^253^^,^^254^^,^^255^

In this light, the mechanistic heterogeneity underlying vitamin D responsiveness challenges the prevailing therapeutic paradigm of universal supplementation targeting arbitrary 25(OH)D thresholds, necessitating a more nuanced approach that addresses tissue-specific metabolic competence. More tractable and potentially more effective approaches may focus on the pathways summarized in the “metabolic vitamin D resistance” model in Figure 2—modulating inflammation, epigenetic regulation, and microcircuit remodeling—using multi-target interventions that restore local responsiveness as well as systemic sufficiency.

Vitamin D research is thus at a useful inflection point. Framing vitamin D as a plastic, context-dependent cellular endocrine network—rather than as a single nutrient or hormone—offers a way to reconcile conflicting trial results, align mechanistic and epidemiologic evidence, and guide the design of next-generation supplementation, sensitization, and delivery strategies. If pursued with appropriate caution and methodological rigor, a microcircuit-based paradigm has the potential to provide a more coherent basis for screening policies, individualized supplementation decisions, and the development of brain-, gut-, or tumor-targeted vitamin D-related interventions.

### Limitations of the study

This review synthesizes mechanistic, translational, and clinical literature to develop a tissue-centered framework for vitamin D biology. It is not a systematic review and did not apply formal risk-of-bias assessment or meta-analytic methods. The proposed concepts, including tissue-specific vitamin D microcircuits and metabolic vitamin D resistance, are intended as integrative and operational frameworks rather than established diagnostic categories. Much of the evidence for microcircuit remodeling derives from *in vitro* systems, animal models, selected human tissues, or disease-specific cohorts, and the extent to which these mechanisms generalize across organs, populations, and clinical settings remains incompletely defined. In addition, several candidate biomarkers discussed here, including tissue-level CYP27B1/CYP24A1 balance, PBMC-based VDR readouts, vitamin D metabolite ratios, and composite microcircuit scores, require analytical standardization and prospective validation before they can guide routine clinical decision-making.

## Acknowledgments

This work was supported by the Military Key Discipline Construction Projects of China under contract number HL21JD1206.

## Author contributions

Conceptualization, B.G. and J.F.; investigation, J.F., J.X., Z.G., H.Z., X.L., and Y.T.; data curation, Z.G. and H.Z.; visualization, J.X. and Y.T.; writing – original draft, J.F.; writing – review and editing, J.X., Z.G., H.Z., X.L., Y.T., and B.G.; supervision, B.G. and Y.T.; project administration, B.G. and Y.T.; funding acquisition, B.G.

## Declaration of interests

The authors declare no competing interests.

## Declaration of generative AI and AI-assisted technologies in the writing process

During the preparation of this work, the authors used ChatGPT in order to improve readability and language. After using this service, the authors reviewed and edited the content as needed and take full responsibility for the content of the published article.

## Supplemental information

Table S1. Exploratory microcircuit-level markers of the vitamin D cellular endocrine system The exploratory markers listed in Table S1 are primarily supported by mechanistic evidence from in vitro experiments, animal models, and limited human studies. They are not standardized for routine clinical use and should be interpreted as hypothesis-generating tools rather than validated endpoints. Evidence levels are qualitative and emphasize the current state of knowledge rather than formal grading. The major unresolved questions highlight potential pathways for translational research and future clinical integration. Abbreviations: DBP, vitamin D-binding protein; CKD, chronic kidney disease; PBMC, peripheral blood mononuclear cell; IBD, inflammatory bowel disease; VDR, vitamin D receptor; VMR, vitamin D metabolite ratio; LC-MS/MS, liquid chromatography-tandem mass spectrometry; RCTs, randomized controlled trials.

## References

[1] Bikle D.D. (2014). Vitamin D metabolism, mechanism of action, and clinical applications. Chem. Biol..

[2] Christakos S., Dhawan P., Verstuyf A., Verlinden L., Carmeliet G. (2016). Vitamin D: metabolism, molecular mechanism of action, and pleiotropic effects. Physiol. Rev..

[3] Chun R.F., Liu P.T., Modlin R.L., Adams J.S., Hewison M. (2014). Impact of vitamin D on immune function: lessons learned from genome-wide analysis. Front. Physiol..

[4] Holick M.F. (2007). Vitamin D deficiency. N. Engl. J. Med..

[5] Liu Y., Zhong Z., Xie J., Ni B., Wu Y. (2025). Neuroprotective roles of vitamin D: bridging the gap between mechanisms and clinical applications in cognitive decline. Int. J. Mol. Sci..

[6] Umar M., Sastry K.S., Chouchane A.I. (2018). Role of vitamin D beyond the skeletal function: a review of the molecular and clinical studies. Int. J. Mol. Sci..

[7] Voiculescu V.M., Nelson Twakor A., Jerpelea N., Pantea Stoian A. (2025). Vitamin d: beyond traditional roles-insights into its biochemical pathways and physiological impacts. Nutrients.

[8] Kojima G., Iliffe S., Tanabe M. (2017). Vitamin d supplementation as a potential cause of u-shaped associations between vitamin d levels and negative health outcomes: a decision tree analysis for risk of frailty. BMC Geriatr..

[9] Liu H., Shen X., Yu T., Wang Y., Cai S., Jiang X., Cai X. (2022). A putative causality of vitamin d in common diseases: a mendelian randomization study. Front. Nutr..

[10] Manson J.E., Cook N.R., Lee I.M., Christen W., Bassuk S.S., Mora S., Gibson H., Gordon D., Copeland T., D’Agostino D. (2019). Vitamin D supplements and prevention of cancer and cardiovascular disease. N. Engl. J. Med..

[11] Pittas A.G., Dawson-Hughes B., Sheehan P., Ware J.H., Knowler W.C., Aroda V.R., Brodsky I., Ceglia L., Chadha C., Chatterjee R. (2019). Vitamin D supplementation and prevention of type 2 diabetes. N. Engl. J. Med..

[12] Liu D., Meng X., Tian Q., Cao W., Fan X., Wu L., Song M., Meng Q., Wang W., Wang Y. (2022). Vitamin d and multiple health outcomes: an umbrella review of observational studies, randomized controlled trials, and mendelian randomization studies. Adv. Nutr..

[13] Wimalawansa S.J., Weiss S.T., Hollis B.W. (2024). Integrating endocrine, genomic, and extra-skeletal benefits of vitamin D into national and regional clinical guidelines. Nutrients.

[14] Ebert R., Jovanovic M., Ulmer M., Schneider D., Meissner-Weigl J., Adamski J., Jakob F. (2004). Down-regulation by nuclear factor kappab of human 25-hydroxyvitamin d3 1alpha-hydroxylase promoter. Mol. Endocrinol..

[15] Tourigny A., Charbonneau F., Xing P., Boukrab R., Rousseau G., St-Arnaud R., Brezniceanu M.L. (2012). CYP24a1 exacerbated activity during diabetes contributes to kidney tubular apoptosis via caspase-3 increased expression and activation. PLoS One.

[16] Nygaard R.H., Nielsen M.C., Antonsen K.W., Højskov C.S., Sørensen B.S., Møller H.J. (2022). Metabolism of 25-hydroxy-vitamin d in human macrophages is highly dependent on macrophage polarization. Int. J. Mol. Sci..

[17] Zehnder D., Bland R., Williams M.C., Mcninch R.W., Howie A.J., Stewart P.M., Hewison M. (2001). Extrarenal expression of 25-hydroxyvitamin D(3)-1 alpha-hydroxylase. J. Clin. Endocrinol. Metab..

[18] Nykjaer A., Fyfe J.C., Kozyraki R., Leheste J.R., Jacobsen C., Nielsen M.S., Verroust P.J., Aminoff M., de la Chapelle A., Moestrup S.K. (2001). Cubilin dysfunction causes abnormal metabolism of the steroid hormone 25(OH) vitamin D(3). Proc. Natl. Acad. Sci. USA.

[19] Nykjaer A., Dragun D., Walther D., Vorum H., Jacobsen C., Herz J., Melsen F., Christensen E.I., Willnow T.E. (1999). An endocytic pathway essential for renal uptake and activation of the steroid 25-(OH) vitamin D3. Cell.

[20] Rowling M.J., Kemmis C.M., Taffany D.A., Welsh J. (2006). Megalin-mediated endocytosis of vitamin d binding protein correlates with 25-hydroxycholecalciferol actions in human mammary cells. J. Nutr..

[21] Shadid I.L.C., Guchelaar H.J., Weiss S.T., Mirzakhani H. (2024). Vitamin D beyond the blood: tissue distribution of vitamin D metabolites after supplementation. Life Sci..

[22] Hewison M. (2010). Vitamin D and the intracrinology of innate immunity. Mol. Cell. Endocrinol..

[23] Zhalehjoo N., Shakiba Y., Panjehpour M. (2017). Gene expression profiles of CYP24a1 and CYP27b1 in malignant and normal breast tissues. Mol. Med. Rep..

[24] Voutsadakis I.A. (2020). Vitamin d receptor (VDR) and metabolizing enzymes CYP27b1 and CYP24a1 in breast cancer. Mol. Biol. Rep..

[25] Adams J.S., Hewison M. (2012). Extrarenal expression of the 25-hydroxyvitamin d-1-hydroxylase. Arch. Biochem. Biophys..

[26] Albiñana C., Zhu Z., Borbye-Lorenzen N., Boelt S.G., Cohen A.S., Skogstrand K., Wray N.R., Revez J.A., Privé F., Petersen L.V. (2023). Genetic correlates of vitamin d-binding protein and 25-hydroxyvitamin d in neonatal dried blood spots. Nat. Commun..

[27] He J., Gao Z., Li X., Zhao L., Tian X., Gao B. (2025). Systematic review of optimizing brain-targeted vitamin d delivery: novel approaches to enhance mental illness therapeutics. Brain Res..

[28] Khan S.S., Petkovich M., Holden R.M., Adams M.A. (2022). Megalin and vitamin d metabolism-implications in non-renal tissues and kidney disease. Nutrients.

[29] Yuan Y., Chen L. (2025). Transporters in vitamin uptake and cellular metabolism: impacts on health and disease. Life Metab..

[30] Leszczyńska D., Szatko A., Latocha J., Kochman M., Duchnowska M., Wójcicka A., Misiorowski W., Zgliczyníski W., Glinicki P. (2024). Persistent hypercalcaemia associated with two pathogenic variants in the CYP24a1 gene and a parathyroid adenoma-a case report and review. Front. Endocrinol..

[31] Reddy G.S., Tserng K.Y. (1989). Calcitroic acid, end product of renal metabolism of 1,25-dihydroxyvitamin D3 through c-24 oxidation pathway. Biochemistry.

[32] Annalora A.J., Goodin D.B., Hong W.X., Zhang Q., Johnson E.F., Stout C.D. (2010). Crystal structure of CYP24a1, a mitochondrial cytochrome p450 involved in vitamin d metabolism. J. Mol. Biol..

[33] Fuchs M.A.A., Grabner A., Shi M., Murray S.L., Burke E.J., Latic N., Thiriveedi V., Roper J., Ide S., Abe K. (2024). Intestinal cyp24a1 regulates vitamin d locally independent of systemic regulation by renal cyp24a1 in mice. J. Clin. Investig..

[34] Jacobs E.T., Van Pelt C., Forster R.E., Zaidi W., Hibler E.A., Galligan M.A., Haussler M.R., Jurutka P.W. (2013). CYP24a1 and CYP27b1 polymorphisms modulate vitamin d metabolism in colon cancer cells. Cancer Res..

[35] Nowacka A., śniegocki M., Bożiłow D., Ziółkowska E.A. (2025). CYP24a1 in small intestinal vitamin d metabolism and clinical implications. Nutrients.

[36] Cappellani D., Brancatella A., Morganti R., Borsari S., Baldinotti F., Caligo M.A., Kaufmann M., Jones G., Marcocci C., Cetani F. (2022). Hypercalcemia due to CYP24a1 mutations: a systematic descriptive review. Eur. J. Endocrinol..

[37] Jarrar Y., Alhammadin G., Lee S.J. (2025). Genetic polymorphisms in cytochrome p450 enzymes involved in vitamin d metabolism and the vitamin d receptor: their clinical relevance. J. Personalized Med..

[38] Doost M.E., Hong J., Broatch J.E., Applegate M.T., Wagner C.E., Marshall P.A., Jurutka P.W. (2024). Synergistic activation of VDR-RXR heterodimers by vitamin d and rexinoids in human kidney and brain cells. Cells.

[39] Pike J.W., Meyer M.B., Lee S.M., Onal M., Benkusky N.A. (2017). The vitamin d receptor: contemporary genomic approaches reveal new basic and translational insights. J. Clin. Investig..

[40] Dimitrov V., Barbier C., Ismailova A., Wang Y., Dmowski K., Salehi-Tabar R., Memari B., Groulx-Boivin E., White J.H. (2021). Vitamin d-regulated gene expression profiles: species-specificity and cell-specific effects on metabolism and immunity. Endocrinology.

[41] Arora J., Wang J., Weaver V., Zhang Y., Cantorna M.T. (2022). Novel insight into the role of the vitamin d receptor in the development and function of the immune system. J. Steroid Biochem. Mol. Biol..

[42] Li Q., Sheikh-Hamad D. (2023). Megalin facilitates the regulation of mitochondrial function by extracellular cues. Integr. Med. Nephrol. Androl..

[43] Pereira F., Fernández-Barral A., Larriba M.J., Barbáchano A., González-Sancho J.M. (2024). From molecular basis to clinical insights: a challenging future for the vitamin d endocrine system in colorectal cancer. FEBS J..

[44] Bikle D.D., Schwartz J. (2019). Vitamin d binding protein, total and free vitamin d levels in different physiological and pathophysiological conditions. Front. Endocrinol..

[45] Speeckaert M., Huang G., Delanghe J.R., Taes Y.E.C. (2006). Biological and clinical aspects of the vitamin d binding protein (gc-globulin) and its polymorphism. Clin. Chim. Acta.

[46] Li L., Han B., Kong Y., Zhang G., Zhang Z. (2024). Vitamin d binding protein in psychiatric and neurological disorders: implications for diagnosis and treatment. Genes Dis..

[47] Lauridsen A.L., Vestergaard P., Nexo E. (2001). Mean serum concentration of vitamin d-binding protein (gc globulin) is related to the gc phenotype in women. Clin. Chem..

[48] Wilson R.T., Bortner J.D., Roff A., Das A., Battaglioli E.J., Richie J.P., Barnholtz-Sloan J., Chinchilli V.M., Berg A., Liu G. (2015). Genetic and environmental influences on plasma vitamin d binding protein concentrations. Transl. Res..

[49] Hendi N.N., Umlai U.K., Albagha O., Nemer G. (2025). Rare-variant genome-wide association and polygenic score assessment of vitamin d status in a middle eastern population. Int. J. Mol. Sci..

[50] Powe C.E., Evans M.K., Wenger J., Zonderman A.B., Berg A.H., Nalls M., Tamez H., Zhang D., Bhan I., Karumanchi S.A. (2013). Vitamin d-binding protein and vitamin d status of black americans and white americans. N. Engl. J. Med..

[51] Saechua C., Sarachana T., Chonchaiya W., Trairatvorakul P., Yuwattana W., Poolcharoen C., Sangritdech M., Saeliw T., van Erp M.L., Sangsuthum S. (2024). Impact of gene polymorphisms involved in the vitamin d metabolic pathway on the susceptibility to and severity of autism spectrum disorder. Sci. Rep..

[52] Beenken A., Cerutti G., Brasch J., Guo Y., Sheng Z., Erdjument-Bromage H., Aziz Z., Robbins-Juarez S.Y., Chavez E.Y., Ahlsen G. (2023). Structures of LRP2 reveal a molecular machine for endocytosis. Cell.

[53] Christensen E.I., Birn H. (2002). Megalin and cubilin: multifunctional endocytic receptors. Nat. Rev. Mol. Cell Biol..

[54] Marzolo M.P., Farfán P. (2011). New insights into the roles of megalin/LRP2 and the regulation of its functional expression. Biol. Res..

[55] Ahuja R., Yammani R., Bauer J.A., Kalra S., Seetharam S., Seetharam B. (2008). Interactions of cubilin with megalin and the product of the amnionless gene (AMN): effect on its stability. Biochem. J..

[56] De S., Kuwahara S., Saito A. (2014). The endocytic receptor megalin and its associated proteins in proximal tubule epithelial cells. Membranes.

[57] Ashley B., Simner C., Manousopoulou A., Jenkinson C., Hey F., Frost J.M., Rezwan F.I., White C.H., Lofthouse E.M., Hyde E. (2022). Placental uptake and metabolism of 25(OH)vitamin d determine its activity within the fetoplacental unit. Elife.

[58] Bikle D.D. (2000).

[59] Vestergaard A.L., Andersen M.K., Andersen H.H., Bossow K.A., Bor P., Larsen A. (2024). Effects of high-dose vitamin d supplementation on placental vitamin d metabolism and neonatal vitamin d status. Nutrients.

[60] Lundgren S., Carling T., Hjälm G., Juhlin C., Rastad J., Pihlgren U., Rask L., Akerström G., Hellman P. (1997). Tissue distribution of human gp330/megalin, a putative Ca(2+)-sensing protein. J. Histochem. Cytochem..

[61] Chapron B., Chapron A., Phillips B., Okoli M.C., Shen D.D., Kelly E.J., Himmelfarb J., Thummel K.E. (2018). Reevaluating the role of megalin in renal vitamin d homeostasis using a human cell-derived microphysiological system. ALTEX.

[62] Hilpert J., Nykjaer A., Jacobsen C., Wallukat G., Nielsen R., Moestrup S.K., Haller H., Luft F.C., Christensen E.I., Willnow T.E. (1999). Megalin antagonizes activation of the parathyroid hormone receptor. J. Biol. Chem..

[63] Chlon T.M., Taffany D.A., Welsh J., Rowling M.J. (2008). Retinoids modulate expression of the endocytic partners megalin, cubilin, and disabled-2 and uptake of vitamin d-binding protein in human mammary cells. J. Nutr..

[64] Gomes J.R., Lobo A., Nogueira R., Terceiro A.F., Costelha S., Lopes I.M., Magalhães A., Summavielle T., Saraiva M.J. (2020). Neuronal megalin mediates synaptic plasticity-a novel mechanism underlying intellectual disabilities in megalin gene pathologies. Brain Commun..

[65] Kong Y., Zhang X., Li L., Zhao T., Huang Z., Zhang A., Sun Y., Jiao J., Zhang G., Liu M. (2025). Microglia-derived vitamin d binding protein mediates synaptic damage and induces depression by binding to the neuronal receptor megalin. Adv. Sci..

[66] Zhang G., Li L., Kong Y., Xu D., Bao Y., Zhang Z., Liao Z., Jiao J., Fan D., Long X. (2024). Vitamin d-binding protein in plasma microglia-derived extracellular vesicles as a potential biomarker for major depressive disorder. Genes Dis..

[67] Bikle D.D. (2021). The free hormone hypothesis: when, why, and how to measure the free hormone levels to assess vitamin d, thyroid, sex hormone, and cortisol status. JBMR Plus.

[68] Chun R.F., Peercy B.E., Orwoll E.S., Nielson C.M., Adams J.S., Hewison M. (2014). Vitamin D and DBP: the free hormone hypothesis revisited. J. Steroid Biochem. Mol. Biol..

[69] Schwartz J.B., Lai J., Lizaola B., Kane L., Weyland P., Terrault N.A., Stotland N., Bikle D. (2014). Variability in free 25(OH) vitamin d levels in clinical populations. J. Steroid Biochem. Mol. Biol..

[70] Tsuprykov O., Buse C., Skoblo R., Haq A., Hocher B. (2018). Reference intervals for measured and calculated free 25-hydroxyvitamin d in normal pregnancy. J. Steroid Biochem. Mol. Biol..

[71] Safadi F.F., Thornton P., Magiera H., Hollis B.W., Gentile M., Haddad J.G., Liebhaber S.A., Cooke N.E. (1999). Osteopathy and resistance to vitamin D toxicity in mice null for vitamin D binding protein. J. Clin. Investig..

[72] Powe C.E., Ricciardi C., Berg A.H., Erdenesanaa D., Collerone G., Ankers E., Wenger J., Karumanchi S.A., Thadhani R., Bhan I. (2011). Vitamin d-binding protein modifies the vitamin d-bone mineral density relationship. J. Bone Miner. Res..

[73] Jassil N.K., Sharma A., Bikle D., Wang X. (2017). Vitamin d binding protein and 25-hydroxyvitamin d levels: emerging clinical applications. Endocr. Pract..

[74] Negri A.L. (2006). Proximal tubule endocytic apparatus as the specific renal uptake mechanism for vitamin d-binding protein/25-(OH)D3 complex. Nephrology.

[75] Bikle D.D., Patzek S., Wang Y. (2018). Physiologic and pathophysiologic roles of extra renal CYP27b1: case report and review. BoneKEy Rep..

[76] Hewison M. (2012). Vitamin d and immune function: autocrine, paracrine or endocrine?. Scand. J. Clin. Lab. Invest. Suppl..

[77] Chauss D., Freiwald T., Mcgregor R., Yan B., Wang L., Nova-Lamperti E., Kumar D., Zhang Z., Teague H., West E.E. (2022). Autocrine vitamin d signaling switches off pro-inflammatory programs of TH1 cells. Nat. Immunol..

[78] Du J., Wei X., Ge X., Chen Y., Li Y.C. (2017). Microbiota-dependent induction of colonic cyp27b1 is associated with colonic inflammation: implications of locally produced 1,25-dihydroxyvitamin d3 in inflammatory regulation in the colon. Endocrinology.

[79] Huang J., Chen T., Liu Y., Lyu L., Li X., Yue W. (2019). How would serum 25(OH)d level change in patients with inflammatory bowel disease depending on intestinal mucosa vitamin d receptor (VDR) and vitamin d1-α hydroxylase (CYP27b1)? Turk. J. Gastroenterol..

[80] Jolliffe D.A., Stefanidis C., Wang Z., Kermani N.Z., Dimitrov V., White J.H., Mcdonough J.E., Janssens W., Pfeffer P., Griffiths C.J. (2020). Vitamin D metabolism is dysregulated in asthma and chronic obstructive pulmonary disease. Am. J. Respir. Crit. Care Med..

[81] Maes K., Serré J., Mathyssen C., Janssens W., Gayan-Ramirez G. (2020). Targeting vitamin d deficiency to limit exacerbations in respiratory diseases: utopia or strategy with potential?. Calcif. Tissue Int..

[82] Osanai M., Lee G.H. (2016). CYP24a1-induced vitamin d insufficiency promotes breast cancer growth. Oncol. Rep..

[83] Jiang Y., Liao L., Li J., Wang L., Xie Z. (2020). Older age is associated with decreased levels of VDR, CYP27b1, and CYP24a1 and increased levels of PTH in human parathyroid glands. Internet J. Endocrinol..

[84] Szymczak-Pajor I., śliwińska A. (2019). Analysis of association between vitamin d deficiency and insulin resistance. Nutrients.

[85] Novakovic B., Sibson M., Ng H.K., Manuelpillai U., Rakyan V., Down T., Beck S., Fournier T., Evain-Brion D., Dimitriadis E. (2009). Placenta-specific methylation of the vitamin d 24-hydroxylase gene: implications for feedback autoregulation of active vitamin d levels at the fetomaternal interface. J. Biol. Chem..

[86] Noyola-Martínez N., Díaz L., Zaga-Clavellina V., Avila E., Halhali A., Larrea F., Barrera D. (2014). Regulation of CYP27b1 and CYP24a1 gene expression by recombinant pro-inflammatory cytokines in cultured human trophoblasts. J. Steroid Biochem. Mol. Biol..

[87] Eyles D.W. (2021). Vitamin d: brain and behavior. JBMR Plus.

[88] Smolders J., Schuurman K.G., Strien M.E.V., Melief J., Hendrickx D., Hol E.M., Eden C.V., Luchetti S., Huitinga I. (2013). Expression of vitamin d receptor and metabolizing enzymes in multiple sclerosis—affected brain tissue. J. Neuropathol. Exp. Neurol..

[89] Mazzetti S., Barichella M., Giampietro F., Giana A., Calogero A.M., Amadeo A., Palazzi N., Comincini A., Giaccone G., Bramerio M. (2022). Astrocytes expressing vitamin d-activating enzyme identify parkinson's disease. CNS Neurosci. Ther..

[90] Savran Z., Baltaci S.B., Aladag T., Mogulkoc R., Baltaci A.K. (2025). Vitamin d and neurodegenerative diseases such as multiple sclerosis (MS), parkinson's disease (PD), alzheimer's disease (AD), and amyotrophic lateral sclerosis (ALS): a review of current literature. Curr. Nutr. Rep..

[91] Keeney J.T., Butterfield D.A. (2015). Vitamin d deficiency and alzheimer disease: common links. Neurobiol. Dis..

[92] Lemoine S., Molin A., Koenig A., Bacchetta J. (2025). Clinical evidence for independent regulation of vitamin d by intestinal CYP24a1. J. Clin. Investig..

[93] Milan K.L., Ramkumar K.M. (2024). Regulatory mechanisms and pathological implications of CYP24a1 in vitamin d metabolism. Pathol. Res. Pract..

[94] Quach H.P., Yang Q.J., Chow E.C., Mager D.E., Hoi S.Y., Pang K.S. (2015). PKPD modelling to predict altered disposition of 1α,25-dihydroxyvitamin d3 in mice due to dose-dependent regulation of CYP27b1 on synthesis and CYP24a1 on degradation. Br. J. Pharmacol..

[95] Yoon S.H., Meyer M.B., Arevalo C., Tekguc M., Zhang C., Wang J.S., Castro Andrade C.D., Strauss K., Sato T., Benkusky N.A. (2023). A parathyroid hormone/salt-inducible kinase signaling axis controls renal vitamin d activation and organismal calcium homeostasis. J. Clin. Investig..

[96] Huang A., Binmahfouz L., Hancock D.P., Anderson P.H., Ward D.T., Conigrave A.D. (2021). Calcium-sensing receptors control CYP27b1-luciferase expression: transcriptional and posttranscriptional mechanisms. J. Endocr. Soc..

[97] Höbaus J., Fetahu I.S., Khorchide M., Manhardt T., Kallay E. (2013). Epigenetic regulation of the 1,25-dihydroxyvitamin d3 24-hydroxylase (CYP24a1) in colon cancer cells. J. Steroid Biochem. Mol. Biol..

[98] Luo W., Karpf A.R., Deeb K.K., Muindi J.R., Morrison C.D., Johnson C.S., Trump D.L. (2010). Epigenetic regulation of vitamin d 24-hydroxylase/CYP24a1 in human prostate cancer. Cancer Res..

[99] Zhou R.H., Li L., Ou Q.J., Wang Y.F., Fang Y.J., Zhang C.X. (2025). CYP24a1 DNA methylation in colorectal cancer as potential prognostic and predictive markers. Biomolecules.

[100] Garcia P.M., Moore J., Kahan D., Hong M.Y. (2020). Effects of vitamin d supplementation on inflammation, colonic cell kinetics, and microbiota in colitis: a review. Molecules.

[101] Al-Nakhle H., Mohsen I., Elnaem B., Alharbi A., Alnakhli I., Almoarfi S., Fallatah J. (2023). Altered expression of vitamin d metabolism genes and circulating micrornas in pbmcs of patients with type 1 diabetes: their association with vitamin d status and ongoing islet autoimmunity. Noncoding. RNA.

[102] Komagata S., Nakajima M., Takagi S., Mohri T., Taniya T., Yokoi T. (2009). Human CYP24 catalyzing the inactivation of calcitriol is post-transcriptionally regulated by mir-125b. Mol. Pharmacol..

[103] Milan K.L., Jayasuriya R., Harithpriya K., Anuradha M., Ramkumar K.M. (2024). MicroRNA-125b regulates vitamin d resistance by targeting CYP24a1 in the progression of gestational diabetes mellitus. J. Steroid Biochem. Mol. Biol..

[104] Wang L., Gao Z., Wang L., Gao Y. (2016). Loss of mir-125b contributes to upregulation of CYP24 in uraemic rats. Nephrology.

[105] Chen G., Kim S.H., King A.N., Zhao L., Simpson R.U., Christensen P.J., Wang Z., Thomas D.G., Giordano T.J., Lin L. (2011). CYP24a1 is an independent prognostic marker of survival in patients with lung adenocarcinoma. Clin. Cancer Res..

[106] Shiratsuchi H., Wang Z., Chen G., Ray P., Lin J., Zhang Z., Zhao L., Beer D., Ray D., Ramnath N. (2017). Oncogenic potential of CYP24a1 in lung adenocarcinoma. J. Thorac. Oncol..

[107] Tannour-Louet M., Lewis S.K., Louet J.F., Stewart J., Addai J.B., Sahin A., Vangapandu H.V., Lewis A.L., Dittmar K., Pautler R.G. (2014). Increased expression of CYP24a1 correlates with advanced stages of prostate cancer and can cause resistance to vitamin D3-based therapies. FASEB J..

[108] Zeng R., Li H., Jia L., Lee S.H., Jiang R., Zhang Y., Hu X., Ye T., Wang X., Yan X. (2022). Association of CYP24a1 with survival and drug resistance in clinical cancer patients: a meta-analysis. BMC Cancer.

[109] Huang W., Ray P., Ji W., Wang Z., Nancarrow D., Chen G., Galbán S., Lawrence T.S., Beer D.G., Rehemtulla A. (2020). The cytochrome p450 enzyme CYP24a1 increases proliferation of mutant KRAS-dependent lung adenocarcinoma independent of its catalytic activity. J. Biol. Chem..

[110] Prosser D.E., Kaufmann M., O'Leary B., Byford V., Jones G. (2007). Single a326g mutation converts human CYP24a1 from 25-OH-d3-24-hydroxylase into -23-hydroxylase, generating 1alpha,25-(OH)2d3-26,23-lactone. Proc. Natl. Acad. Sci. USA.

[111] Taban I.M., Zhu J., Deluca H.F., Simons C. (2017). Analysis of the binding sites of vitamin d 1α-hydroxylase (CYP27b1) and vitamin D 24-hydroxylase (CYP24a1) for the design of selective CYP24a1 inhibitors: homology modelling, molecular dynamics simulations and identification of key binding requirements. Bioorg. Med. Chem..

[112] Sawyer C.W., Tuey S.M., West R.E., Nolin T.D., Joy M.S. (2022). Physiologically based pharmacokinetic modeling of vitamin D(3) and metabolites in vitamin d-insufficient patients. Drug Metab. Dispos..

[113] Wang T.T., Nestel F.P., Bourdeau V., Nagai Y., Wang Q., Liao J., Tavera-Mendoza L., Lin R., Hanrahan J.W., Mader S., White J.H. (2004). Cutting edge: 1,25-dihydroxyvitamin d3 is a direct inducer of antimicrobial peptide gene expression. J. Immunol..

[114] Gombart A.F., Borregaard N., Koeffler H.P. (2005). Human cathelicidin antimicrobial peptide (CAMP) gene is a direct target of the vitamin d receptor and is strongly up-regulated in myeloid cells by 1,25-dihydroxyvitamin d3. FASEB J..

[115] Liu P.T., Stenger S., Li H., Wenzel L., Tan B.H., Krutzik S.R., Ochoa M.T., Schauber J., Wu K., Meinken C. (2006). Toll-like receptor triggering of a vitamin d-mediated human antimicrobial response. Science.

[116] Yuk J.M., Shin D.M., Lee H.M., Yang C.S., Jin H.S., Kim K.K., Lee Z.W., Lee S.H., Kim J.M., Jo E.K. (2009). Vitamin d3 induces autophagy in human monocytes/macrophages via cathelicidin. Cell Host Microbe.

[117] Yong Y.Y., Zhang L., Hu Y.J., Wu J.M., Yan L., Pan Y.R., Tang Y., Yu L., Law B.Y.K., Yu C.L. (2022). Targeting autophagy regulation in NLRP3 inflammasome-mediated lung inflammation in COVID-19. Clin. Immunol..

[118] Cantorna M.T. (2006). Vitamin d and its role in immunology: multiple sclerosis, and inflammatory bowel disease. Prog. Biophys. Mol. Biol..

[119] Ferreira G.B., Gysemans C.A., Demengeot J., da Cunha J.P.M.C.M., Vanherwegen A.S., Overbergh L., Van Belle T.L., Pauwels F., Verstuyf A., Korf H., Mathieu C. (2014). 1,25-dihydroxyvitamin d3 promotes tolerogenic dendritic cells with functional migratory properties in NOD mice. J. Immunol..

[120] Kong J., Zhang Z., Musch M.W., Ning G., Sun J., Hart J., Bissonnette M., Li Y.C. (2008). Novel role of the vitamin d receptor in maintaining the integrity of the intestinal mucosal barrier. Am. J. Physiol. Gastrointest. Liver Physiol..

[121] Sun J., Zhang Y.G. (2022). Vitamin d receptor influences intestinal barriers in health and disease. Cells.

[122] Li Y., Guo Y., Geng C., Song S., Yang W., Li X., Wang C. (2024). Vitamin d/vitamin d receptor protects intestinal barrier against colitis by positively regulating notch pathway. Front. Pharmacol..

[123] Chen X.q., Mao J.y., Wang C.s., Li W.b., Han T.t., Lv K., Li J.n. (2022). CYP24a1 involvement in inflammatory factor regulation occurs via the wnt signaling pathway. Curr. Med. Sci..

[124] Hansdottir S., Monick M.M., Hinde S.L., Lovan N., Look D.C., Hunninghake G.W. (2008). Respiratory epithelial cells convert inactive vitamin d to its active form: potential effects on host defense. J. Immunol..

[125] Chen H., Lu R., Zhang Y.g., Sun J. (2018). Vitamin d receptor deletion leads to the destruction of tight and adherens junctions in lungs. Tissue Barriers.

[126] Bikle D.D. (2011). Vitamin D metabolism and function in the skin. Mol. Cell. Endocrinol..

[127] Bikle D.D. (2012). Vitamin D and the skin: physiology and pathophysiology. Rev. Endocr. Metab. Disord..

[128] Relhan V., Goel K., Kochhar A., Garg V., Wadhwa B. (2015). Vitamin D and skin diseases: a review. Indian J. Dermatol. Venereol. Leprol..

[129] Sailike B., Onzhanova Z., Akbay B., Tokay T., Molnár F. (2024). Vitamin d in central nervous system: implications for neurological disorders. Int. J. Mol. Sci..

[130] Pietruszkiewicz J., Mrozek K., Zwierz M., Wińska A., Suprunowicz M., Oracz A.J., Waszkiewicz N. (2025). The neuroprotective potential of vitamin D(3). Nutrients.

[131] Wang W., Li Y., Meng X. (2023). Vitamin D and neurodegenerative diseases. Heliyon.

[132] Faye P.A., Poumeaud F., Miressi F., Lia A.S., Demiot C., Magy L., Favreau F., Sturtz F.G. (2019). Focus on 1,25-dihydroxyvitamin d3 in the peripheral nervous system. Front. Neurosci..

[133] Cui X., Eyles D.W. (2022). Vitamin D and the central nervous system: causative and preventative mechanisms in brain disorders. Nutrients.

[134] Chen Z., Yuan Z., Yang S., Zhu Y., Xue M., Zhang J., Leng L. (2023). Brain energy metabolism: astrocytes in neurodegenerative diseases. CNS Neurosci. Ther..

[135] Won S., Sayeed I., Peterson B.L., Wali B., Kahn J.S., Stein D.G. (2015). Vitamin d prevents hypoxia/reoxygenation-induced blood-brain barrier disruption via vitamin d receptor-mediated NF-kb signaling pathways. PLoS One.

[136] Huang Y., Chen Y., Wu Y., Wu Y., Dai X., Feng J., Li X. (2025). Association of vitamin d with risk of dementia: a dose-response meta-analysis of observational studies. Front. Neurol..

[137] Hummel D.M., Fetahu I.S., Gröschel C., Manhardt T., Kállay E. (2014). Role of proinflammatory cytokines on expression of vitamin d metabolism and target genes in colon cancer cells. J. Steroid Biochem. Mol. Biol..

[138] Schrumpf J.A., van der Does A.M., Hiemstra P.S. (2020). Impact of the local inflammatory environment on mucosal vitamin d metabolism and signaling in chronic inflammatory lung diseases. Front. Immunol..

[139] Bao B.Y., Ting H.J., Hsu J.W., Yasmin-Karim S., Messing E., Lee Y.F. (2010). Down-regulation of NF-kappab signals is involved in loss of 1alpha,25-dihydroxyvitamin d3 responsiveness. J. Steroid Biochem. Mol. Biol..

[140] Chung C., Silwal P., Kim I., Modlin R.L., Jo E.K. (2020). Vitamin d-cathelicidin axis: at the crossroads between protective immunity and pathological inflammation during infection. Immune Netw..

[141] Grivennikov S., Karin E., Terzic J., Mucida D., Yu G.Y., Vallabhapurapu S., Scheller J., Rose-John S., Cheroutre H., Eckmann L., Karin M. (2009). IL-6 and stat3 are required for survival of intestinal epithelial cells and development of colitis-associated cancer. Cancer Cell.

[142] Li Y., de Haar C., Chen M., Deuring J., Gerrits M.M., Smits R., Xia B., Kuipers E.J., van der Woude C.J. (2010). Disease-related expression of the IL6/STAT3/SOCS3 signalling pathway in ulcerative colitis and ulcerative colitis-related carcinogenesis. Gut.

[143] Gröschel C., Tennakoon S., Kállay E. (2015). Cytochrome p450 vitamin d hydroxylases in inflammation and cancer. Adv. Pharmacol..

[144] Jeon S.M., Shin E.A. (2018). Exploring vitamin d metabolism and function in cancer. Exp. Mol. Med..

[145] Gasperini B., Falvino A., Piccirilli E., Tarantino U., Botta A., Visconti V.V. (2023). Methylation of the vitamin d receptor gene in human disorders. Int. J. Mol. Sci..

[146] Saccone D., Asani F., Bornman L. (2015). Regulation of the vitamin D receptor gene by environment, genetics and epigenetics. Gene.

[147] Deeb K.K., Luo W., Karpf A.R., Omilian A.R., Bshara W., Tian L., Tangrea M.A., Morrison C.D., Johnson C.S., Trump D.L. (2011). Differential vitamin d 24-hydroxylase/CYP24a1 gene promoter methylation in endothelium from benign and malignant human prostate. Epigenetics.

[148] Li C., Chen Y., Zhu H., Zhang X., Han L., Zhao Z., Wang J., Ning L., Zhou W., Lu C. (2020). Inhibition of histone deacetylation by MS-275 alleviates colitis by activating the vitamin d receptor. J. Crohns Colitis.

[149] Gerbeth L., Glauben R. (2021). Histone deacetylases in the inflamed intestinal epithelium-promises of new therapeutic strategies. Front. Med..

[150] Marangoni K., Dorneles G., Da Silva D.M., Pinto L.P., Rossoni C., Fernandes S.A. (2023). Diet as an epigenetic factor in inflammatory bowel disease. World J. Gastroenterol..

[151] Gallagher J.C. (2013). Vitamin D and aging. Endocrinol Metab. Clin. N. Am..

[152] Veldurthy V., Wei R., Oz L., Dhawan P., Jeon Y.H., Christakos S. (2016). Vitamin d, calcium homeostasis and aging. Bone Res..

[153] Martinelli R.P., Rayego-Mateos S., Alique M., Márquez-Expósito L., Tejedor-Santamaria L., Ortiz A., González-Parra E., Ruiz-Ortega M. (2023). Vitamin d, cellular senescence and chronic kidney diseases: what is missing in the equation?. Nutrients.

[154] Niculescu D.A., Deacu L.G., Caragheorgheopol A., Popescu N., Ghemigian A., Procopiuc C., Rosca R., Poiana C. (2021). Combined effects of vitamin d status, renal function and age on serum parathyroid hormone levels. Front. Endocrinol..

[155] Odera K., Goto S., Takahashi R. (2007). Age-related change of endocytic receptors megalin and cubilin in the kidney in rats. Biogerontology.

[156] Laird E., O’Halloran A.M., Molloy A.M., Healy M., Bourke N., Kenny R.A. (2023). Vitamin d status & associations with inflammation in older adults. PLoS One.

[157] Carlberg C., Velleuer E. (2024). Vitamin d and aging: central role of immunocompetence. Nutrients.

[158] Fantini C., Corinaldesi C., Lenzi A., Migliaccio S., Crescioli C. (2023). Vitamin d as a shield against aging. Int. J. Mol. Sci..

[159] Hyppönen E., Vimaleswaran K.S., Zhou A. (2022). Genetic determinants of 25-hydroxyvitamin d concentrations and their relevance to public health. Nutrients.

[160] Simpson C.A., Zhang J.H., Vanderschueren D., Fu L., Pennestri T.C., Bouillon R., Cole D.E.C., Carpenter T.O. (2020). Relationship of total and free 25-hydroxyvitamin d to biomarkers and metabolic indices in healthy children. J. Clin. Endocrinol. Metab..

[161] Karcıoğlu Batur L., özaydın A., Maviş M.E., Gürsu G.G., Harbige L., Hekim N. (2021). Vitamin-d binding protein gene polymorphisms and serum 25-hydroxyvitamin-d in a turkish population. Metabolites.

[162] Aloia J., Mikhail M., Dhaliwal R., Shieh A., Usera G., Stolberg A., Ragolia L., Islam S. (2015). Free 25(OH)d and the vitamin d paradox in african americans. J. Clin. Endocrinol. Metab..

[163] Mokry L.E., Ross S., Ahmad O.S., Forgetta V., Smith G.D., Leong A., Greenwood C.M.T., Thanassoulis G., Richards J.B., Richards J.B. (2015). Vitamin d and risk of multiple sclerosis: a mendelian randomization study. PLoS Med..

[164] Manousaki D., Harroud A., Mitchell R.E., Ross S., Forgetta V., Timpson N.J., Smith G.D., Polychronakos C., Richards J.B. (2021). Vitamin D levels and risk of type 1 diabetes: a mendelian randomization study. PLoS Med..

[165] Lawler T., Warren Andersen S. (2023). Serum 25-hydroxyvitamin d and cancer risk: a systematic review of mendelian randomization studies. Nutrients.

[166] Fang A., Zhao Y., Yang P., Zhang X., Giovannucci E.L. (2024). Vitamin d and human health: evidence from mendelian randomization studies. Eur. J. Epidemiol..

[167] Revez J.A., Lin T., Qiao Z., Xue A., Holtz Y., Zhu Z., Zeng J., Wang H., Sidorenko J., Kemper K.E. (2020). Genome-wide association study identifies 143 loci associated with 25 hydroxyvitamin D concentration. Nat. Commun..

[168] Dhull R.S., Jain R., Deepthi B., Cheong H.I., Saha A., Mehndiratta M., Basu S. (2020). Vitamin d-dependent rickets (VDDR) type 1: case series of two siblings with a CYP27b1 mutation and review of the literature. J. Bras. Nefrol..

[169] Zamanfar D., Ghazaiean M. (2023). An overview of CYP27b1 enzyme mutation and management: a case report and review of the literature. Clin. Case Rep..

[170] Pilz S., Theiler-Schwetz V., Pludowski P., Zelzer S., Meinitzer A., Karras S.N., Misiorowski W., Zittermann A., März W., Trummer C. (2022). Hypercalcemia in pregnancy due to CYP24a1 mutations: case report and review of the literature. Nutrients.

[171] Kang S.J., Lee R., Kim H.S. (2019). Infantile hypercalcemia with novel compound heterozygous mutation in SLC34a1 encoding renal sodium-phosphate cotransporter 2a: a case report. Ann. Pediatr. Endocrinol. Metab..

[172] Bizerea-Moga T.O., Chisavu F., Ilies C., Olah O., Marginean O., Gafencu M., Doros G., Stroescu R. (2023). Phenotype of idiopathic infantile hypercalcemia associated with the heterozygous pathogenic variant of SLC34a1 and CYP24a1. Children-Basel.

[173] Meza-Meza M.R., Vizmanos B., Rivera-Escoto M., Ruiz-Ballesteros A.I., Pesqueda-Cendejas K., Parra-Rojas I., Montoya-Buelna M., Luquín S., Campos-López B., Mora-García P.E. (2022). Vitamin D receptor (VDR) genetic variants: relationship of foki genotypes with VDR expression and clinical disease activity in systemic lupus erythematosus patients. Genes.

[174] Nunes I.F.O.C., Cavalcante A.A.C.M., Alencar M.V.O.B., Carvalho M.D.F., Sarmento J.L.R., Teixeira N.S.C.C.A., Paiva A.A., Carvalho L.R., Nascimento L.F.M., Cruz M.S.P. (2020). Meta-analysis of the association between the rs228570 vitamin d receptor gene polymorphism and arterial hypertension risk. Adv. Nutr..

[175] Alvarez-Hernandez D., Naves-Diaz M., Gomez-Alonso C., Coto E., Cannata-Andia J.B. (2008). Tissue-specific effect of VDR gene polymorphisms on the response to calcitriol. J. Nephrol..

[176] Uitterlinden A.G., Fang Y., Bergink A.P., van Meurs J.B.J., van Leeuwen H.P.T.M., Pols H.A.P. (2002). The role of vitamin d receptor gene polymorphisms in bone biology. Mol. Cell. Endocrinol..

[177] Pérez-Alonso M., Briongos L., Ruiz-Mambrilla M., Velasco E.A., Olmos J.M., de Luis D., Dueñas-Laita A., Pérez-Castrillón J. (2020). Association between bat vitamin d receptor 3' haplotypes and vitamin d levels at baseline and a lower response after increased vitamin d supplementation and exposure to sunlight. Int. J. Vitam. Nutr. Res..

[178] Usategui-Martín R., De Luis-Román D.A., Fernández-Gómez J.M., Ruiz-Mambrilla M., Pérez-Castrillón J.L. (2022). Vitamin d receptor (vdr) gene polymorphisms modify the response to vitamin D supplementation: a systematic review and meta-analysis. Nutrients.

[179] Giustina A., Bilezikian J.P., Adler R.A., Banfi G., Bikle D.D., Binkley N.C., Bollerslev J., Bouillon R., Brandi M.L., Casanueva F.F. (2024). Consensus statement on vitamin D status assessment and supplementation: whys, whens, and hows. Endocr. Rev..

[180] Chun R.F., Shieh A., Gottlieb C., Yacoubian V., Wang J., Hewison M., Adams J.S. (2019). Vitamin d binding protein and the biological activity of vitamin D. Front. Endocrinol..

[181] Yu C., Xue H., Wang L., Chen Q., Chen X., Zhang Y., Hu G., Ling W. (2018). Serum bioavailable and free 25-hydroxyvitamin d levels, but not its total level, are associated with the risk of mortality in patients with coronary artery disease. Circ. Res..

[182] Rivera-Paredez B., Hidalgo-Bravo A., León-Reyes G., León-Maldonado L.S., Aquino-Gálvez A., Castillejos-López M., Denova-Gutiérrez E., Flores Y.N., Salmerón J., Velázquez-Cruz R. (2021). Total, bioavailable, and free 25-hydroxyvitamin d equally associate with adiposity markers and metabolic traits in mexican adults. Nutrients.

[183] Schwartz J.B., Lai J., Lizaola B., Kane L., Markova S., Weyland P., Terrault N.A., Stotland N., Bikle D. (2014). A comparison of measured and calculated free 25(OH) vitamin D levels in clinical populations. J. Clin. Endocrinol. Metab..

[184] Yamshchikov A.V., Kurbatova E.V., Kumari M., Blumberg H.M., Ziegler T.R., Ray S.M., Tangpricha V. (2010). Vitamin D status and antimicrobial peptide cathelicidin (LL-37) concentrations in patients with active pulmonary tuberculosis. Am. J. Clin. Nutr..

[185] Zhao S., He Y., Pan M., Chen B., Zhang S., Zhang Y., Zhu Y. (2022). Expression and significance of serum vitamin d and LL-37 levels in infants with bacterial pneumonia. Front. Pediatr..

[186] Deng C., Wu Y. (2025). Vitamin d-parathyroid hormone-fibroblast growth factor 23 axis and cardiac remodeling. Am. J. Cardiovasc. Drugs.

[187] Latic N., Erben R.G. (2021). FGF23 and vitamin d metabolism. JBMR Plus.

[188] Shardlow A., Mcintyre N.J., Fluck R.J., Mcintyre C.W., Taal M.W. (2017). Associations of fibroblast growth factor 23, vitamin d and parathyroid hormone with 5-year outcomes in a prospective primary care cohort of people with chronic kidney disease stage 3. BMJ Open.

[189] Underland L., Markowitz M., Gensure R. (2020). Calcium and phosphate hormones: vitamin d, parathyroid hormone, and fibroblast growth factor 23. Pediatr. Rev..

[190] Aldekwer S., Goncalves-Mendes N., Bingula R., Martinroche G., Lanchais K., Rougé S., Farges M.C., Rossary A., Diab-Assaf M., Vasson M.P., Talvas J. (2022). 25-hydroxyvitamin d potentializes extracellular cathelicidin release from human PBMC stimulated ex vivo with either bacterial (LPS) or viral (p: IC) mimetics. J. Physiol. Biochem..

[191] Law S.P.L., Gatt P.N., Schibeci S.D., Mckay F.C., Vucic S., Hart P., Byrne S.N., Brown D., Stewart G.J., Liddle C. (2021). Expression of CYP24a1 and other multiple sclerosis risk genes in peripheral blood indicates response to vitamin d in homeostatic and inflammatory conditions. Gene Immun..

[192] Smolders J., Thewissen M., Theunissen R., Peelen E., Knippenberg S., Menheere P., Cohen Tervaert J.W., Hupperts R., Damoiseaux J. (2011). Vitamin d-related gene expression profiles in immune cells of patients with relapsing remitting multiple sclerosis. J. Neuroimmunol..

[193] Enlund-Cerullo M., Koljonen L., Holmlund-Suila E., Hauta-Alus H., Rosendahl J., Valkama S., Helve O., Hytinantti T., Viljakainen H., Andersson S. (2019). Genetic variation of the vitamin d binding protein affects vitamin d status and response to supplementation in infants. J. Clin. Endocrinol. Metab..

[194] Gibbs D.C., Barry E.L., Fedirko V., Baron J.A., Bostick R.M. (2023). Impact of common vitamin d-binding protein isoforms on supplemental vitamin d3 and/or calcium effects on colorectal adenoma recurrence risk: a secondary analysis of a randomized clinical trial. JAMA Oncol..

[195] Newton D.A., Baatz J.E., Kindy M.S., Gattoni-Celli S., Shary J.R., Hollis B.W., Wagner C.L. (2019). Vitamin d binding protein polymorphisms significantly impact vitamin d status in children. Pediatr. Res..

[196] Sollid S.T., Hutchinson M.Y.S., Berg V., Fuskevåg O.M., Figenschau Y., Thorsby P.M., Jorde R. (2016). Effects of vitamin d binding protein phenotypes and vitamin d supplementation on serum total 25(OH)d and directly measured free 25(OH)d. Eur. J. Endocrinol..

[197] Yao P., Sun L., Lu L., Ding H., Chen X., Tang L., Xu X., Liu G., Hu Y., Ma Y. (2017). Effects of genetic and nongenetic factors on total and bioavailable 25(OH)d responses to vitamin D supplementation. J. Clin. Endocrinol. Metab..

[198] Fu L., Yun F., Oczak M., Wong B.Y.L., Vieth R., Cole D.E.C. (2009). Common genetic variants of the vitamin d binding protein (DBP) predict differences in response of serum 25-hydroxyvitamin d [25(OH)d] to vitamin d supplementation. Clin. Biochem..

[199] Gopal-Kothandapani J.S., Evans L.F., Walsh J.S., Gossiel F., Rigby A.S., Eastell R., Bishop N.J. (2019). Effect of vitamin d supplementation on free and total vitamin d: a comparison of asians and caucasians. Clin. Endocrinol..

[200] Schoenmakers I., Jones K.S. (2017). Letter to the editor: the effect of genetic factors on the response to vitamin d supplementation may be mediated by vitamin d-binding protein concentrations. J. Clin. Endocrinol. Metab..

[201] Yang F., Sun M., Sun C., Li J., Yang X., Bi C., Wang M., Pu L., Wang J., Wang C. (2020). Associations of c-reactive protein with 25-hydroxyvitamin d in 24 specific diseases: a cross-sectional study from NHANES. Sci. Rep..

[202] Zhou A., Hyppönen E. (2023). Vitamin d deficiency and c-reactive protein: a bidirectional mendelian randomization study. Int. J. Epidemiol..

[203] Krajewska M., Witkowska-Sędek E., Rumińska M., Stelmaszczyk-Emmel A., Sobol M., Majcher A., Pyrżak B. (2022). Vitamin d effects on selected anti-inflammatory and pro-inflammatory markers of obesity-related chronic inflammation. Front. Endocrinol..

[204] Ramezani R., Ghorbaninejad P., Eslahi M., Sheikhi L., Abbasi F., Hasanzadeh M., Mohammadpour S., Milajerdi A. (2024). Effects of vitamin d supplementation on serum 25-hydroxy cholecalciferol in inflammatory bowel diseases: a meta-analysis of randomized clinical trials. Int. J. Prev. Med..

[205] Yu Y., Tian L., Xiao Y., Huang G., Zhang M. (2018). Effect of vitamin d supplementation on some inflammatory biomarkers in type 2 diabetes mellitus subjects: a systematic review and meta-analysis of randomized controlled trials. Ann. Nutr. Metab..

[206] Dozio E., Briganti S., Vianello E., Dogliotti G., Barassi A., Malavazos A.E., Ermetici F., Morricone L., Sigruener A., Schmitz G., Corsi Romanelli M.M. (2015). Epicardial adipose tissue inflammation is related to vitamin d deficiency in patients affected by coronary artery disease. Nutr. Metabol. Cardiovasc. Dis..

[207] Hahn J.M., Combs K.A., Phillips C.M., Warner P.M., Qazi U.A., Powell H.M., Supp D.M. (2025). CYP24a1 is overexpressed in keloid keratinocytes and its inhibition alters profibrotic gene expression. Burns Trauma.

[208] Luo W., Johnson C.S., Trump D.L. (2016). Vitamin d signaling modulators in cancer therapy. Vitam. Horm..

[209] Schuster I., Egger H., Herzig G., Reddy G.S., Schmid J.A., Schüssler M., Vorisek G. (2006). Selective inhibitors of vitamin d metabolism--new concepts and perspectives. Anticancer Res..

[210] Aggeletopoulou I., Kalafateli M., Geramoutsos G., Triantos C. (2024). Recent advances in the use of vitamin d organic nanocarriers for drug delivery. Biomolecules.

[211] Wang K., Yang R., Li J., Wang H., Wan L., He J. (2025). Nanocarrier-based targeted drug delivery for alzheimer's disease: addressing neuroinflammation and enhancing clinical translation. Front. Pharmacol..

[212] Chaykovska L., Heunisch F., von Einem G., Alter M.L., Hocher C.F., Tsuprykov O., Dschietzig T., Kretschmer A., Hocher B. (2016). Urinary vitamin d binding protein and kim-1 are potent new biomarkers of major adverse renal events in patients undergoing coronary angiography. PLoS One.

[213] Semnani-Azad Z., Wang W.Z.N., Cole D.E.C., Johnston L.W., Wong B.Y.L., Fu L., Retnakaran R., Harris S.B., Hanley A.J. (2024). Urinary vitamin d binding protein: a marker of kidney tubular dysfunction in patients at risk for type 2 diabetes. J. Endocr. Soc..

[214] Jiménez-Sousa M.Á., Martínez I., Medrano L.M., Fernández-Rodríguez A., Resino S. (2018). Vitamin d in human immunodeficiency virus infection: influence on immunity and disease. Front. Immunol..

[215] Abboud M., Rizk R., Alanouti F., Papandreou D., Haidar S., Mahboub N. (2020). The health effects of vitamin d and probiotic co-supplementation: a systematic review of randomized controlled trials. Nutrients.

[216] Aggeletopoulou I., Marangos M., Assimakopoulos S.F., Mouzaki A., Thomopoulos K., Triantos C. (2023). Vitamin d and microbiome: molecular interaction in inflammatory bowel disease pathogenesis. Am. J. Pathol..

[217] Pagnini C., Di Paolo M.C., Graziani M.G., Delle Fave G. (2021). Probiotics and vitamin d/vitamin d receptor pathway interaction: potential therapeutic implications in inflammatory bowel disease. Front. Pharmacol..

[218] Singh P., Rawat A., Alwakeel M., Sharif E., Al Khodor S. (2020). The potential role of vitamin d supplementation as a gut microbiota modifier in healthy individuals. Sci. Rep..

[219] Dai Q., Zhu X., Manson J.E., Song Y., Li X., Franke A.A., Costello R.B., Rosanoff A., Nian H., Fan L. (2018). Magnesium status and supplementation influence vitamin d status and metabolism: results from a randomized trial. Am. J. Clin. Nutr..

[220] Liu Y., Gong R., Ma H., Chen S., Sun J., Qi J., Pang Y., An J., Su Z. (2022). Dietary magnesium intake level modifies the association between vitamin d and insulin resistance: a large cross-sectional analysis of american adults. Front. Nutr..

[221] Kellermann L., Hansen S.L., Maciag G., Granau A.M., Johansen J.V., Teves J.M., Bressan R.B., Pedersen M.T., Soendergaard C., Baattrup A.M. (2024). Influence of vitamin d receptor signalling and vitamin d on colonic epithelial cell fate decisions in ulcerative colitis. J. Crohns Colitis.

[222] Sosa-Díaz E., Reyes-Gopar H., de Anda-Jáuregui G., Hernández-Lemus E. (2025). Single-cell analysis dissects the effects of vitamin d on genetic senescence signatures across murine tissues. Nutrients.

[223] Zhang J., Wang J., Zhou Q., Chen Z., Zhuang J., Zhao X., Gan Z., Wang Y., Wang C., Molday R.S. (2025). Spatiotemporally resolved transcriptomics reveals the cellular dynamics of human retinal development. Nat. Commun..

[224] Kojima H., Itoh T. (2020). Preparation and use of turn-on fluorescent probe for detection and live cell imaging of vitamin d receptor as a target protein. STAR Protoc..

[225] Hendi N.N., Nemer G. (2025). SDR42e1 modulates vitamin d absorption and cancer pathogenesis: insights from an in vitro model. Front. Endocrinol..

[226] Bhimavarapu U., Battineni G., Chintalapudi N. (2025). Machine learning-driven prediction of vitamin d deficiency severity with hybrid optimization. Bioengineering-Basel.

[227] Guo J., He Q., Li Y. (2024). Machine learning-based prediction of vitamin d deficiency: NHANES 2001-2018. Front. Endocrinol..

[228] Jiménez-Gaona Y., Vivanco-Galván O., Castillo-Malla D., Vivanco-Gualán I., Díaz-Guzmán P. (2025). VITA-d: a radiomic web tool for predicting vitamin d deficiency levels. Appl. Sci.-Basel.

[229] Dugar A., Hoofnagle A.N., Sanchez A.P., Ward D.M., Corey-Bloom J., Cheng J.H., Ix J.H., Ginsberg C. (2023). The vitamin d metabolite ratio (vmr) is a biomarker of vitamin d status that is not affected by acute changes in vitamin d binding protein. Clin. Chem..

[230] Wise S.A., Hahm G., Burdette C.Q., Tai S.S.C., Camara J.E., Sempos C.T., Williams E.L. (2023). Determination of 24,25-dihydroxyvitamin D(3) in vitamin d external quality assessment scheme samples using a reference measurement procedure. J. Steroid Biochem. Mol. Biol..

[231] Bouillon R., Manousaki D., Rosen C., Trajanoska K., Rivadeneira F., Richards J.B. (2022). The health effects of vitamin d supplementation: evidence from human studies. Nat. Rev. Endocrinol..

[232] Jørgensen H.S., Vervloet M., Cavalier E., Bacchetta J., de Borst M.H., Bover J., Cozzolino M., Ferreira A.C., Hansen D., Herrmann M. (2025). The role of nutritional vitamin d in chronic kidney disease-mineral and bone disorder in children and adults with chronic kidney disease, on dialysis, and after kidney transplantation-a european consensus statement. Nephrol. Dial. Transplant..

[233] Huynh N., Vonmoss L., Smith D., Rahman I., Felemban M.F., Zuo J., Rody W.J., Mchugh K.P., Holliday L.S. (2016). Characterization of regulatory extracellular vesicles from osteoclasts. J. Dent. Res..

[234] Negri M., Amatrudo F., Gentile A., Patalano R., Montò T., de Angelis C., Simeoli C., Pirchio R., Auriemma R.S., Colao A. (2022). Vitamin d reverts the exosome-mediated transfer of cancer resistance to the mtor inhibitor everolimus in hepatocellular carcinoma. Front. Oncol..

[235] Hanel A., Carlberg C. (2020). Vitamin D and evolution: pharmacologic implications. Biochem. Pharmacol..

[236] Kollitz E.M., Zhang G., Hawkins M.B., Whitfield G.K., Reif D.M., Kullman S.W. (2016). Evolutionary and functional diversification of the vitamin d receptor-lithocholic acid partnership. PLoS One.

[237] Krist A.H., Davidson K.W., Mangione C.M., Cabana M., Caughey A.B., Davis E.M., Donahue K.E., Doubeni C.A., Epling J.W., Tseng C.W. (2021). Screening for vitamin d deficiency in adults: us preventive services task force recommendation statement. JAMA.

[238] Lapauw B., Laurent M.R., Rozenberg S., Body J.J., Bruyère O., Gielen E., Goemaere S., Iconaru L., Cavalier E. (2024). When and how to evaluate vitamin d status? A viewpoint from the belgian bone club. Nutrients.

[239] Floreskul V., Juma F.Z., Daniel A.B., Zamir I., Rawdin A., Stevenson M., Mughal Z., Padidela R. (2020). Cost-effectiveness of vitamin d supplementation in pregnant woman and young children in preventing rickets: a modeling study. Front. Public Health.

[240] Lacey L.F., Armstrong D.J., Royle E., Magee P., Pourshahidi L.K., Ray S., Strain J.J., Mcsorley E. (2022). Cost-effectiveness of vitamin D(3) supplementation in older adults with vitamin d deficiency in ireland. BMJ Nutr. Prev. Health.

[241] Lee R.H., Weber T., Colón-Emeric C. (2013). Comparison of cost-effectiveness of vitamin d screening with that of universal supplementation in preventing falls in community-dwelling older adults. J. Am. Geriatr. Soc..

[242] Bakker E., Hendrikse N.M., Ehmann F., van der Meer D.S., Llinares Garcia J., Vetter T., Starokozhko V., Mol P.G.M. (2022). Biomarker qualification at the european medicines agency: a review of biomarker qualification procedures from 2008 to 2020. Clin. Pharmacol. Ther..

[243] Bakker E., Starokozhko V., Kraaijvanger J.W.M., Heerspink H.J.L., Mol P.G.M. (2023). Precision medicine in regulatory decision making: biomarkers used for patient selection in european public assessment reports from 2018 to 2020. Clin. Transl. Sci..

[244] Morris H.A., Anderson P.H. (2010). Autocrine and paracrine actions of vitamin d. Clin. Biochem. Rev..

[245] Voltan G., Cannito M., Ferrarese M., Ceccato F., Camozzi V. (2023). Vitamin D: an overview of gene regulation, ranging from metabolism to genomic effects. Genes.

[246] Janubová M., žitňanová I. (2024). The effects of vitamin d on different types of cells. Steroids.

[247] Lin Y., Chen J., Xin S., Lin Y., Chen Y., Zhou X., Chen H., Li X. (2024). CYP24a1 affected macrophage polarization through degradation of vitamin d as a candidate biomarker for ovarian cancer prognosis. Int. Immunopharmacol..

[248] Nemeth Z., Patonai A., Simon-Szabó L., Takács I. (2023). Interplay of vitamin d and sirt1 in tissue-specific metabolism-potential roles in prevention and treatment of non-communicable diseases including cancer. Int. J. Mol. Sci..

[249] Sîrbe C., Rednic S., Grama A., Pop T.L. (2022). An update on the effects of vitamin d on the immune system and autoimmune diseases. Int. J. Mol. Sci..

[250] Grant W., Wimalawansa S., Pludowski P., Cheng R. (2025). Vitamin d: evidence-based health benefits and recommendations for population guidelines. Nutrients.

[251] Kämpe A., Enlund-Cerullo M., Valkama S., Holmlund-Suila E., Rosendahl J., Hauta-Alus H., Pekkinen M., Andersson S., Mäkitie O. (2019). Genetic variation in GC and CYP2r1 affects 25-hydroxyvitamin d concentration and skeletal parameters: a genome-wide association study in 24-month-old finnish children. PLoS Genet..

[252] Speeckaert M.M., Speeckaert R., Delanghe J.R. (2021). The biologic importance of the vitamin d binding protein polymorphism in pediatric COVID-19 patients. Eur. J. Pediatr..

[253] Argano C., Torres A., Orlando V., Cangialosi V., Maggio D., Pollicino C., Corrao S. (2025). Molecular insight into the role of vitamin D in immune-mediated inflammatory diseases. Int. J. Mol. Sci..

[254] El Miedany Y., Toth M., Mohamed El Gaafary M., A Mahran S., Hassan W., Hassan Abu-Zaid M., Elwakil W., Basyoni Selim W., Ahmed Sultan E., Saber Ibraheem G., Galal Moussa S. (2025). Vitamin d management update: evidence-based guidelines for vitamin d optimization by the egyptian academy for bone and muscle health. Egypt. Rheumatol. Rehabil..

[255] Saad M.A., Sathyam D., Koora S., Kottireddy S. (2025). Vitamin D supplementation in deficiency states and combined calcium-vitamin D therapy in diabetes prevention and management: a systematic review of clinical evidence. Cureus.

