# Supplementary material for: Vitamin D as a cellular endocrine system: Tissue-specific microcircuits, immune reprogramming, and metabolic resistance
Source: iScience. 2026 Jun 1;29(6):116160. doi: 10.1016/j.isci.2026.116160 (PMC13253088; doi:10.1016/j.isci.2026.116160)
Supplement: Table S1. Exploratory microcircuit-level markers of the vitamin D cellular endocrine system — The exploratory markers listed in Table S1 are primarily supported by mechanistic evidence from in vitro experiments, animal models, and limited human studies. They are not standardized for routine clinical use and should be interpreted as hypothesis-generating tools rather than validated endpoints. Evidence levels are qualitative and emphasize the current state of knowledge rather than formal grading. The major unresolved questions highlight potential pathways for translational research and future clinical integration. Abbreviations: DBP, vitamin D-binding protein; CKD, chronic kidney disease; PBMC, peripheral blood mononuclear cell; IBD, inflammatory bowel disease; VDR, vitamin D receptor; VMR, vitamin D metabolite ratio; LC-MS/MS, liquid chromatography-tandem mass spectrometry; RCTs, randomized controlled trials. [file mmc1.pdf]

iScience, Volume *29*

Supplemental information

Vitamin D as a cellular endocrine system: Tissue-specific microcircuits, immune reprogramming, and metabolic resistance

Jialiang Feng, Jun Xiao, Zhiyuan Gao, Hui Zhou, Xilian Li, Yini Tian, and Biao Gao

Table S1. Exploratory microcircuit-level markers of the vitamin D cellular endocrine system, related to Figure 1 and Table 1

Pathway Component	Marker	Key Evidence Types	Evidence Nature	Measurement and Availability	Major Unresolved Questions
Transport / Tissue Level	Urinary DBP, urinary C-megalin (proximal tubule pathway)	CKD, diabetic nephropathy, small intervention studies; functional work on megalin-mediated reabsorption	Mechanistic emphasis / limited clinical	Specific ELISA or mass spectrometry; research only; no reference ranges	In general populations or mild renal impairment, can urinary DBP/C-megalin sensitively reflect megalin–DBP–25(OH)D recycling and identify supplementation-resistant individuals?
Transport / Tissue Level	Tissue megalin/cubilin expression (kidney, placenta, intestine, mammary gland, choroid plexus)	Tissue arrays, immunohistochemistry; animal models	Mechanistic	Requires tissue specimens and pathology platforms; research only	To what extent does down-regulation in different organs/disease stages cause "tissue-level vitamin D deficiency"? Are there blood/urine surrogate markers?
Activation (CYP27B1)	Tissue CYP27B1 mRNA/protein (intestine, lung, placenta, brain, skin)	Extensive in vitro/animal studies; limited human biopsies	Mechanistic	Requires tissue samples + molecular assays; research only	How do organ-specific CYP27B1 levels dynamically respond to circulating 25(OH)D, inflammation, and aging? Is there a reversible "reprogramming window" amenable to intervention?
Activation (CYP27B1)	PBMC CYP27B1 baseline and ex vivo induction	Infection/autoimmune cohorts; TLR–CYP27B1–cathelicidin in vitro studies	Mechanistic	PBMC isolation + transcript/protein assays; research only	Can PBMC CYP27B1 serve as a simplified readout of immune "vitamin D responsiveness"? What is the magnitude of intra- and inter-day variability versus technical error?
Inactivation (CYP24A1)	Tissue or PBMC CYP24A1 expression; CYP24A1/CYP27B1 ratio	Tumor, IBD, renal tissue studies; animal/in vitro experiments	Mechanistic	Requires tissue or PBMC; research only	Under which inflammatory/tumor contexts does high CYP24A1 represent "metabolic resistance" versus non-catalytic oncogenic/protective roles? Could the ratio evolve into a reproducible "microcircuit state score"?
Receptor (VDR)	Tissue VDR expression (skeletal muscle, kidney, parathyroid, immune cells, brain)	Immunohistochemistry, transcriptomics; animal models; limited human samples	Mechanistic	Requires tissue; research only	What is the quantitative relationship between tissue VDR levels and functional state? In aging/chronic inflammation, are epigenetic and post-translational modifications reversible, and how can they be targeted?
Receptor (VDR)	PBMC VDR protein/mRNA	In vitro $1,25(OH)_2D$ stimulation; small patient cohorts	Mechanistic	PBMC isolation + molecular assays	To what extent do PBMC VDR levels represent receptor status in key target organs (gut, lung, brain)? Are they independently associated with outcomes or supplementation response?
Receptor (VDR)	Common VDR polymorphisms/haplotypes (FokI, BsmI, ApaI, TaqI)	Large cohorts and case–control studies; subgroup analyses in supplementation trials; limited functional work	Clinical evidence limited / functional weak	Genotyping mature but not routine in clinics	What is the "gain coefficient" of VDR polymorphisms for different organs/outcomes? Could multi-locus scoring help identify individuals requiring higher doses or special formulations?
Downstream Functional Response	Antimicrobial peptide cathelicidin/LL-37 induction	TLR–CYP27B1–cathelicidin axis in vitro/animal models; infection/autoimmune cohorts	Mechanistic	mRNA/protein quantification; research only	Can LL-37 induction be used as a functional marker of vitamin D–mediated immune responsiveness, especially in populations with recurrent infection, chronic lung disease, or immunosuppression? Could it be incorporated as a mechanistic endpoint in intervention trials?
Downstream Functional Response	PBMC or tissue VDR target gene transcriptome ("vitamin D response signature")	Extensive in vitro $1,25(OH)_2D$ stimulation + transcriptomics; limited patient PBMC studies	Mechanistic	Requires high-throughput omics platforms; costly; not yet simplified	Can a small set of genes be distilled from the full transcriptome to create a simplified "vitamin D response score" applicable in large cohorts, with proven incremental predictive value, test–retest reliability, and cross-platform consistency?
Integrated Metabolism and Microcircuit Phenotype	"Vitamin D metabolome" (multi-metabolite profiling)	LC-MS/MS quantification of multiple metabolites; pattern analyses in high-dose supplementation trials	Mechanistic	Advanced mass spectrometry feasible but expensive and complex; research use only	Can a stable, reproducible "vitamin D metabolic fingerprint" be defined to characterize individual flux and microcircuit phenotype? Does it truly provide stratification or predictive value in RCTs?
Integrated Metabolism and Microcircuit Phenotype	Composite "microcircuit score" (integrating VMR, CYP27B1/CYP24A1, VDR target genes, etc.)	Conceptual framework; partial analyses in selected disease cohorts	Exploratory	Still at the stage of exploratory analyses; no standardized format	How can a simple, reproducible "microcircuit score" be constructed and validated without excessive testing burden? At what level should it be positioned in guidelines and clinical pathways (research tool *vs.* decision-making aid)?

Note: The exploratory markers listed in Table S1 are primarily supported by mechanistic evidence from in vitro experiments, animal models, and limited human studies. They are not standardized for routine clinical use and should be interpreted as hypothesis-generating tools rather than validated endpoints. Evidence levels are qualitative and emphasize the current state of knowledge rather than formal grading. The major unresolved questions highlight potential pathways for translational research and future clinical integration.
